# Preparation, Spectroscopic Characterization, Theoretical Investigations, and In Vitro Anticancer Activity of Cd(II), Ni(II), Zn(II), and Cu(II) Complexes of 4(3*H*)-Quinazolinone-Derived Schiff Base

**DOI:** 10.3390/molecules25245973

**Published:** 2020-12-16

**Authors:** Ubale Panchsheela Ashok, Shiva Prasad Kollur, Nishad Anil, Bansode Prakash Arun, Sanjay Namdev Jadhav, Sanjay Sarsamkar, Vasant Baburao Helavi, Asha Srinivasan, Sandeep Kaulage, Ravindra Veerapur, Sarah Al-Rashed, Asad Syed, Joaquín Ortega-Castro, Juan Frau, Norma Flores-Holguín, Daniel Glossman-Mitnik

**Affiliations:** 1 Department of Chemistry, Rajaram College, Shivaji University, Kolhapur 416 004, Maharashtra, India; panchsheela_ubale@rediffmail.com; 2 N.K. Orchid College of Engineering and Technology, Solapur 413 002, Maharashtra, India; 3 Department of Sciences, Amrita School of Arts and Sciences, Amrita Vishwa Vidyapeetham, Mysuru campus, Mysuru 570 026, Karnataka, India; 4 Department of Chemistry, Institute of Science, Mumbai 400 032, Maharashtra, India; anilnishad789@gmail.com; 5 Department of Chemistry, Sangola College Sangola, Sangola 413 307, Maharashtra, India; bansode.prakash4@gmail.com; 6 Organic Chemistry Division, CSIR-National Chemical Laboratory, Pune 411 008, Maharashtra, India; sanjay.jadhav31@gmail.com; 7 Department of Chemistry, Walchand Institute of Technology, Solapur 413 002, Maharashtra, India; sarsamkar30@yahoo.co.in; 8 Division of Nanoscience and Technology, Faculty of Life Sciences, JSS Academy of Higher Education and Research, Mysuru 570 015, Karnataka, India; asha.srinivasan@jssuni.edu.in; 9 Department of Chemistry, Indian Institute of Science, Education and Research, Pune 411 008, Maharashtra, India; sandeep.kaulage@gmail.com; 10 Department of Materials and Metallurgy Engineering, Malawi Institute of Technology, Malawi University of Science and Technology, P.O. Box 5916, Limbe, Malawi; rveerapur@must.ac.mw; 11 Department of Botany and Microbiology, College of Science, King Saud University, P.O. Box 2455, Riyadh 11451, Saudi Arabia; salrashed@ksu.edu.sa (S.A.-R.); assyed@ksu.edu.sa (A.S.); 12 Departament de Química, Universitat de les IllesBalears, 07122 Palma de Mallorca, Spain; joaquin.castro@uib.es (J.O.-C.); juan.frau@uib.es (J.F.); daniel.glossman@cimav.edu.mx (D.G.-M.); 13 Laboratorio Virtual NANOCOSMOS, Departamento de Medio Ambiente y Energía, Centro de Investigación en MaterialesAvanzados, Chihuahua, Chih 31136, Mexico; norma.flores@cimav.edu.mx

**Keywords:** 3-quinolin-4(3*H)*-one, metal complexes, spectral studies, chemical reactivity properties, conceptual DFT

## Abstract

Herein, we report the synthesis and characterization of a new Schiff base ligand 3-[[(*E*)-(3-hydroxyphenyl*)-*methylidene]amino]*-2-*methyl*-*quinazolin*-*4*(*3*H)-*one (HAMQ) and its Cd(II), Ni(II), Zn(II), and Cu(II) complexes (**C_1_**–**C_4_**). The ligand HAMQ was synthesized by reacting 3-hydroxybenzaldehyde and 3-amino-2-methyl-4(3*H)-*quinazolinone in a 1:1 molar ratio. The structure of the ligand and its complexes (**C_1_**–**C_4_**) were evaluated using ultraviolet (UV)–visible (Vis) light spectroscopy, ^1^H-NMR, Fourier-transform infrared (FT-IR) spectroscopy, MS, elemental analysis, conductance data, and thermogravimetric analysis (TGA). The characterization results suggested that the bidentate ligand, HAMQ, coordinated to the metal center through the lactum oxygen and the azomethine nitrogen. Moreover, all the metal complexes were analyzed using powder X-ray diffraction studies, which revealed that all of them belong to a triclinic crystal system. The research was supplemented by density functional theory (DFT) studies on the IR and UV–Vis spectra, as well as the chemical reactivity of the HAMQ and its four metallic derivatives making use of conceptual density functional theory (CDFT) by means of KID (Koopmans in DFT) methodology. The synthesized complexes displayed significant in vitro anticancer activity against human cancer cell lines (HeLa and HCT-115).

## 1. Introduction

Cancer has been a major cause of mortality in recent years. Based on the origin and location of the secondary tumor, radiation or chemotherapy is generally advised. The scattered dose in radiation and the nonspecificity in chemotherapy are general limitations in cancer therapies [1,2]. These techniques have a few disadvantages, for example, deadly results, antagonistic medication responses, significant expense, high repeat rates, and treatment disappointment [3]. Owing to these drawbacks, the breakthrough discovery of cisplatin by Rosenberg et al. opened a new era of utilization of metal complexes in anticancer medication [4]. Although most of the marketed anticancer drugs are essentially organic compounds or natural products, metal complexes are recently gaining much attention in oncology due to their unique and broad-spectrum pharmacological activities. Schiff bases provide useful applications to tune their anticancer activity due to substitution of various moieties in their aromatic ring [5]. The applications of Schiff bases for the synthesis of privileged frameworks are of prime significance in diversity-oriented synthesis and medicinal chemistry, among others. The phenolato ligands of metal complexes show highly potent in vitro anticancer activities [6,7].

Recently, transition metal complexes of quinazoline derivatives have been generally investigated for the versatility in terms of their exquisite colors, coordination geometries, spectroscopic properties, technical applications depending on the molecular study, and their mysterious biological processes. These complexes can exhibit antibacterial, anticonvulsant, anti-inflammatory, hypnotic, antimalarial, and antihypertensive properties [8,9,10,11]. In recent years, considerable research has focused on the synthesis and properties of metal complexes of mixed ligands since they can furnish new materials with paramount applications such as electrical conductivity [12], nonlinear optical properties [13], magnetic exchange [14,15], photoluminescence [16], and antimicrobial activity [17,18,19,20,21]. Schiff bases as ligands with their transition metal complexes are of predominant scientific interest because of their multiple ramifications.

In light of the above discussion and in continuation of our work on the advancement of new anticancer agents, herein, we report the synthesis and characterization of Cd(II), Ni(II), Zn(II), and Cu(II) complexes using a Schiff base ligand. The research was further supported by performing computational density functional theory (DFT) studies on the Fourier-transform infrared (FT-IR) and ultraviolet–visible light (UV–Vis) spectra, as well as the chemical reactivity of 3-[[(*E*)-(3-hydroxyphenyl*)-*methylidene]amino]*-2-*methyl*-*quinazolin*-*4*(*3*H)-*one *(*HAMQ) and its complexes by means of conceptual density functional theory (CDFT) through the ”Koopmans in DFT” (KID) approximation. Furthermore, the synthesized complexes were screened for their in vitro anticancer activity against human cancer cell lines, HeLa and HCT-115, using the sulforhodamine B (SRB) assay.

## 2. Results and Discussion

**Scheme 1:**
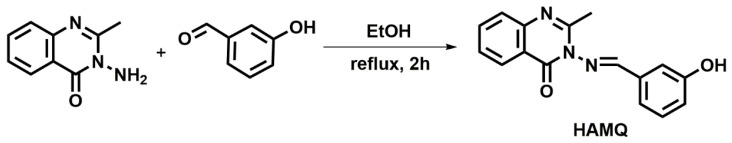
General route for the synthesis of 3-[[(*E*)-(3-hydroxyphenyl*)-*methylidene]amino]*-2-*methyl*-*quinazolin*-*4*(*3*H)-*one (HMAQ) ligand.

HAMQ was obtained according to Scheme 1. The proposed structural details were elucidated based on UV–Vis, FT-IR, ^1^H-NMR, and mass spectral analysis. The analytical and physical data of metal complexes are presented in Table S1 (Supplementary Materials).

### 2.1. ^1^H-NMR Spectral Analysis

The ^1^H-NMR spectra of compounds were recorded in DMSO-d_6_ to explain the structure of HAMQ and its Cd(II) and Zn(II) metal complexes (Figures S1–S3, Supplementary Materials). The ^1^H-NMR spectra of the **C**_1_ and **C_3_** complexes showed resonance of ligand overlap to the protons of 1,10-phenantroline. However, a broad multiplet observed in the range δ 6.84–8.61 ppm for **C_1_** and **C_3_** complexes was assigned to the phenyl group of 1,10-phenanthroline together with the ring proton of HAMQ. The peak that appeared at δ 9.06 ppm in the ligand was due to the azomethine proton group shifted to δ 8.63 and 8.64 ppm in the two metal complexes, respectively. In the ligand, the peak at δ 10.62 ppm was due to the hydroxy proton and was assigned between δ 10.01 and 10.11 ppm in the metal complexes. The spectra of the complexes exhibited a broad singlet around δ 2.55 ppm due to the –CH_3_ proton of HAMQ. Furthermore, ^13^C-NMR evidenced the presence of the azomethine group at δ 169.81.57, as well as other signals related to methyl carbon at δ 22.67 and aromatic carbons at δ 116.47–134.62 (Figures S4 and S5, Supplementary Materials).

### 2.2. FT-IR Spectral Studies

FT-IR spectra of the **HAMQ** and its metal complexes (**C_1_**–**C_3_**) were compared on the basis of any changes in the bands during coordination. The comparison of the infrared spectral data of the ligand and its complexes confirmed that complexation occurred, as significant shifts were observed in the bands of the azomethine group υ(CH=N) and lactum υ(C=O) group (Figures S6–S8 and Table S2, Supplementary Materials). The FT-IR spectrum of HAMQ showed the expected characteristic imine band at 1610 cm^−1^, shifted in the region 1523–1589 cm^−1^ in metal complexes due to metal coordination. A sharp band in the ligand at 1655 cm^−1^ due to υ(C=O) stretching was shifted to lower frequencies in complexes in the region 1605–1643 cm^−1^. Moreover, this was also supported by the appearance of the weak bands of υ(M–O) and υ(M–N), which were observed in the ranges of 533–540 cm^−1^ and 422–442 cm^−1^, respectively. The simulated FT-IR spectra for **HAMQ** and its four metal complexes (**C_1_**–**C_4_**) calculated as mentioned in Section 2.2 are displayed in Figure S9 (Supplementary Materials).

### 2.3. UV–Visible Spectroscopy

In order to investigate the photophysical properties of HAMQ and its complexes (**C_1_**–**C_4_**), UV–visible spectroscopic studies were carried out. The absorption spectra of HAMQ and its complexes (**C_1_**–**C_4_**) were recorded in DMSO solution, and the optical characteristics in terms of maximum absorption wavelength (λ_max_) and molar absorptivity epsilon (ε) values are provided in Table S3 (Supplementary Materials). The molar absorptivity epsilon (ε) values corresponding to 33,557 and 33,222 M^−1^·cm^−1^ in the electronic spectrum of HAMQ suggested the presence of *π* → π^*^ and *n* → π^*^ transitions within the aromatic moiety and quinazoline ring. Similarly, another band observed at 32,573 M^−1^·cm^−1^ could be due to the *n* → π* transitions of the C=O (carbonyl group) of HAMQ. Since the cadmium ion has *d*^10^ electronic configuration, there were no *d*–*d* electron transitions observed as the *d*-orbitals are completely filled. The Ni(II) complex (**C_2_**) showed an absorption band at epsilon (ε) = 14,493 M^−1^·cm^−1^ assigned to 3A_2g_ → 3T_g_(F) and 3A_2g_(F) → 1T_g_(F), corresponding to octahedral geometry. The diamagnetic Zn(II) complex (**C_3_**) showed electronic absorption bands at epsilon (ε) = 14,749 and 14,085 M^−1^·cm^−1^ attributed to intra-ligand transitions predicting octahedral geometry. The electronic absorption spectrum of Cu(II) complex showed a *d*–*d* band at ε = 14,045 M^−1^·cm^−1^ which was assigned to the 2E_g_ → 2T_g_ transition (Figure S10, Supplementary Materials). This suggested the distorted octahedral geometry of **C_4_** [22]. The simulated UV–Vis spectra for the ligand and its metal complexes are presented in Figure S11 (Supplementary Materials), displaying several theoretically allowed transitions between different orbitals. Among them, those corresponding to the maximum-wavelength absorption, generally associated with a highest occupied molecular orbital (HOMO)–lowest unoccupied molecular orbital (LUMO) transition were shown to correlate well with the experimental UV–vis spectra of the metal complexes displayed in Figure S10 (Supplementary Materials).

### 2.4. Mass Spectral Studies

The formation of HAMQ and its metal complexes (**C_1_**–**C_4_**) was elucidated by mass spectroscopic analysis. The molecular ion peak of HAMQ was observed at *m*/*z* = 281.1119, which was attributed to the [M + H] adduct of the reported ligand. Similarly, the peaks observed at *m*/*z* = 591.0194, 594.8981, and 592.3096 corresponded to the mass values of **C_2_**, **C_3_**, and **C_4_**, respectively. Furthermore, all the experimental mass values were in good agreement with the theoretically calculated values (Figures S12–S15, Supplementary Materials).

### 2.5. Thermal Analysis

All the obtained complexes were investigated using TGA and DTA studies (Figure S16, Supplementary Materials). Table S4 (Supplementary Materials) summarizes the recorded TGA/DTA curves of all the complexes. The **C_1_** complex underwent a two-step decomposition process. The initial weight loss occurring in the temperature range of 26–250 °C corresponded to the loss of chloride ions. The organic ligand moiety further decomposed at the temperature range of 250–900 °C leaving a final stable residue of CdO. The **C_2_** complex underwent a two-step decomposition process. The first step involved the loss of chloride ions in the range of 26–220 °C. The organic ligand moiety decomposed exothermically in the range 220–430 °C, pertaining to the loss of C_6_H_4_, while nitrogen was lost in the range of 430–596 °C. The final residual fraction corresponded to the formation of thermally stable NiO. In a similar manner, **C_3_** and **C_4_** complexes also exhibited two-step decomposition processes. The initial weight losses occurred in the low temperature ranges of 26–380 °C for **C_3_** and 26–200 °C for **C_4_**, indicating the loss of H_2_O molecules, while the second step occurred due to the loss of **HAMQ** with two coordinated chloro ligands in the temperature ranges of 380–815 °C and 200–800 °C for **C_3_** and **C_4_** metal complexes, leaving behind stable residues of CuO and ZnO, respectively. The results of TGA/DTA studies showed good agreement with the formulas of synthesized complexes derived from the analytical data.

#### X-ray Diffraction (XRD) Analysis

The most useful tool to obtain information regarding the structure of complexes is single-crystal X-ray crystallography. Due to the difficulty in obtaining crystalline complexes in proper symmetry, X-ray powder diffraction patterns were obtained for **C_1_**–**C_4_** metal complexes using the X-pert High Score Plus software in the range of 10–80° with steps of 0.0200°. X-ray powder diffraction analysis is a useful tool to determine the type of crystal system, as well as the lattice parameters and the cell volume. The XRD patterns indicated the crystalline nature of all the complexes, as manifested in Figure S17 (Supplementary Materials). For all complexes, *d* values, full width at half maximum (FWHM), and relative intensities are listed in Tables S5–S8 (Supplementary Materials). From the indexed data, the unit cell parameters, volume, and average crystallite size were also calculated and are tabulated in Table 1. The powder XRD patterns of all the complexes were found to represent a triclinic crystal system. The full width at half maximum (FWHM) of the diffraction peaks obtained from the refinement was used to calculate the particle size. The average size of the samples was calculated with the help of the Debye–Scherrer equation,

(1) $$D=\frac{0.90\lambda }{\beta \mathrm{Cos}\theta },$$

where λ is the wavelength (Cu Kα), β is the full width at half maximum (FWHM), and θ is the diffraction angle. The diffraction peaks indicated that the synthesized materials were in the nanometer range.

### 2.6. In Vitro Anticancer Activity Evaluation Using Sulforhodamine-B (SRB) Assay

**Figure 1:**
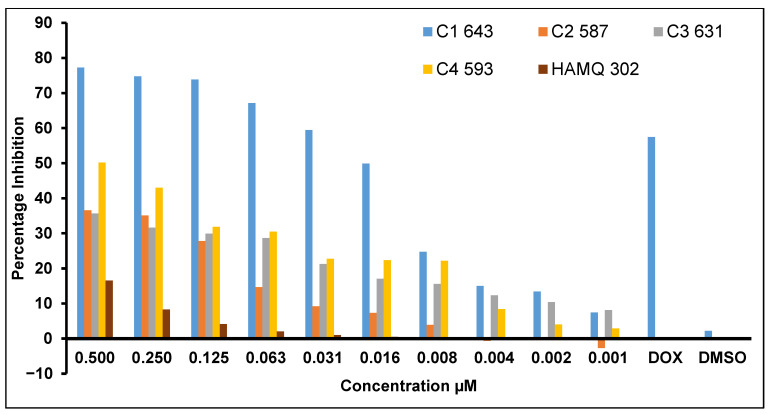
Sulforhodamine B colorimetric assay in colorectal cancer cell culture (HCT115) to analyze the percentage inhibition for all complexes. Doxorubicin was used as a positive control, while dimethyl sulfoxide (DMSO) was used as a vehicle control.

Metal-based compounds hold extraordinary promise in anticancer drug discovery. Recently, many transition metal complexes demonstrated a broad-spectrum and outstanding anticancer activity profile against a variety of malignancies [23]. Therefore, in the current study, we examined the in vitro anticancer activity of the synthesized complexes against human cancer cervical cell line HeLa and human colon cancer cell line HCT-115 by utilizing the sulforhodamine B (SRB) assay after 72 h exposure [24]. The results revealed that the ligand **HAMQ** was found to show cytotoxicity against the tested cancer cell lines (HCT115), with an IC_50_ value of 16.56 µM corresponding to 0.5 µg/mL. It is noteworthy that most of the synthesized complexes demonstrated a weak antiproliferative effect when tested on tested human colon cancer cells (HCT 115) compared to the positive control (standard drug, doxorubicin, 18.39 µM). However, the synthesized complexes slowed the proliferation of tested cancer cell lines in a dose-dependent manner. The outcomes clearly demonstrated the cell-type-specific antiproliferative activity for the metal complexes when treated with HCT-115 and HeLa cancer cells. Furthermore, it is worth mentioning that, even at low concentration (0.000976 µg/mL, IC_50_ = 0.032 µM), all four complexes under investigation exhibited significant antiproliferative activity when compared to the standard drug doxorubicin (IC_50_ = 18.39 µM). The most active complex **C_1_** displayed greater anticancer activity at a concentration ranging from 0.01952–7.77 µg/mL. The IC_50_ value of **C_1_** was 0.97 µM, corresponding to a concentration of 0.0626 µg/mL (Table S9, Supplementary Materials) (Figure 1). Complex **C_3_** also displayed anticancer activity with an IC_50_ value of 1.98 µM corresponding to a concentration of 0.125 µg/mL. Likewise, complexes **C_2_** and **C_4_** had IC_50_ values of 1.06 and 1.05 µM at a concentration of 0.625 µg/mL, respectively (Table S9, Supplementary Materials).

**Figure 2:**
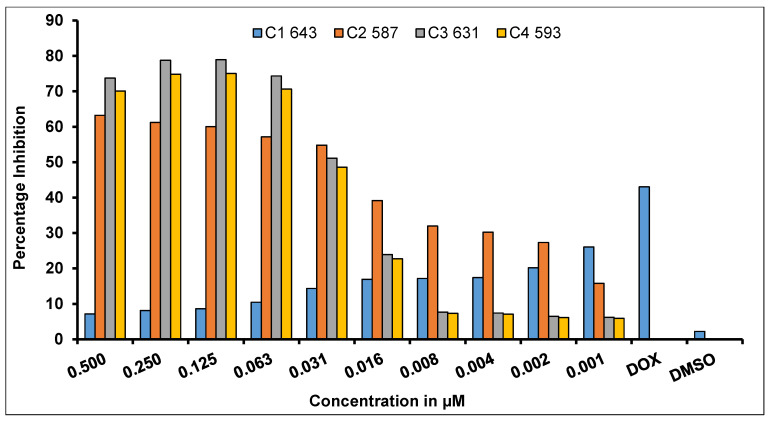
Sulforhodamine B (SRB) colorimetric assay in HeLa cell line (cervical cancer) culture to analyze percentage inhibition for all complexes. Doxorubicin was used as a positive control, while DMSO was used as a vehicle control.

In HeLa cell lines (human cervical cancer cells), the metal complexes showed different distinctive results when compared to HCT115 colorectal cancer cell lines. Complexes **C_2_**, **C_3_**, and **C_4_** displayed higher antiproliferative activity when treated with human cervical cancer HeLa cells with a percentage cell growth inhibition of approximately 75% in a wide concentration range encompassing 0.5, 0.25, 0.125, and 0.0625 µg/mL (Figure 2), which was even higher than standard drug doxorubicin (1 µg/mL). On the other hand, complex **C_1_** displayed comparatively lower antiproliferative activity at both low and high concentrations, which might have been because of cell line resistance to **C_1_**, on three independent occasions (*n* = 3). Similarly, the cytotoxicity of quinazoline Schiff bases against HeLa cells, which were found to be relatively less cytotoxic (IC_50_ = 4 to >250 µM), was reported (Table 2). Thus, our examinations suggest the potential utilization of the synthesized compounds as anticancer agents, subject to further careful investigations including in vivo or animal model experiments, as well as pharmacokinetic and pharmacodynamic studies.

### 2.7. Determination of the Descriptors Related to Chemical Reactivity

**Figure 3:**
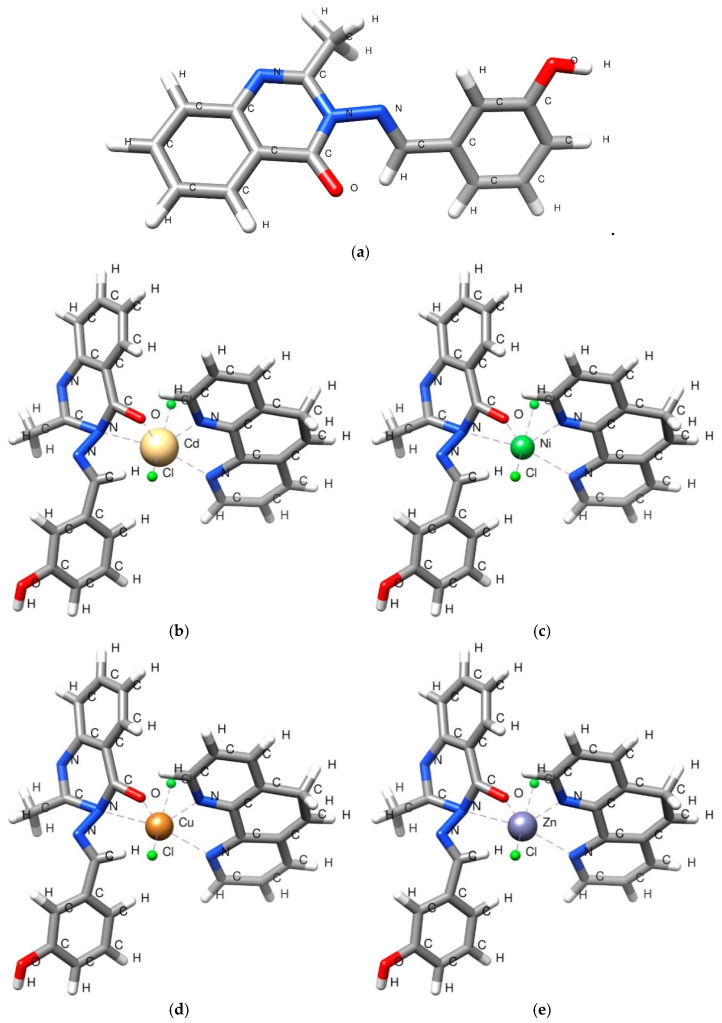
Optimized structures of (**a**) **HMAQ**, as well as (**b**) **C_1_** (**c**) **C_2_**, (**d**) **C_3_**, and (**e**) **C_4_** complexes.

The optimized molecular structures of the ligand and its four metallic complexes, determined as outlined in Section 3.2, are shown in the form of graphical sketches in Figure 3.

The main objective behind this part of the study was the evaluation of the fulfillment of the KID protocol for the systems under study. To this end, several KID quantities based on the HOMO and LUMO calculations were associated with the values of the vertical I and A following the ΔSCF methodology, where SCF refers to the self-consistent field procedure. These three quantities or descriptors are connected to the Koopman theorem by relating $\varepsilon_{H}$ to −I, $\varepsilon_{L}$ to −A, and the HOMO–LUMO gap through the following definitions: $J_{I}=\;\left|\varepsilon_{H}+E_{gs}\left(N-1\right)-E_{gs}\left(N\right)\right|$, $J_{A}=\;\left|\varepsilon_{L}+E_{gs}\left(N\right)-E_{gs}\left(N+1\right)\right|$, and $J_{HL}=\sqrt{J_{I}^{2}+J_{A}^{2}}$. The $J_{A}$ descriptor is only valid if the HOMO energy of the radical anion (the singly occupied molecular orbital (SOMO)) is approximately close to the LUMO energy of the neutral system. As a complement, another descriptor ΔSL was developed by the CIMAV-UIB group [25,26,27,28,29,30,31,32,33,34], for a further verification of the accuracy of the employed methodology. The corresponding values of this part of the study are shown in Table 3.

It can be seen from the results in Table 2 that the MN12SX/Def2TZVP/DMSO model chemistry employed for the determination of the electronic properties of the molecular systems under study was validated in consideration of the KID methodology. This implies that the chemical reactivity descriptors could be calculated directly by means of the HOMO and LUMO values of the ground state obtained for these molecular systems.

Having validated the KID methodology considered in previous studies using finite difference approximation, the definitions given in the Supplementary Materials can be used to estimate the global reactivity descriptors [35,36,37,38]. The results for the ligand **HAMQ** and its four metal complexes are shown in Table 4.

From the analysis of the results in Table 4, three main conclusions may be considered. Firstly, the electron-donating power was larger than the electron-accepting power for the compounds, which is an expected result considering their electronic structures. Secondly, the HAMQ ligand was slightly more reactive than the corresponding metallic complexes when considering the values of the global hardness, because this property is a direct measure of the deformation of the electron density and, hence, of the chemical reactivity. The final remark is that the chemical reactivity for the Cd(II), Ni(II), and Zn(II) complexes was approximately on the same order, albeit somewhat lower than that for the Cu(II) complex. Indeed, these conclusions regarding the chemical reactivity of these compounds could be of interest for studies on their potential therapeutic abilities.

## 3. Experimental

### 3.1. Materials and Methods

3-Amino-2-methyl-4(*3H*)-quinazolinone was purchased from Sigma Aldrich (CA, USA) and was used as received. 3-Hydroxybenzaldehyde, metal salts, and solvents were commercially available in high purity and used as received. The completion of the reaction was monitored by spotting the reaction mixture using thin-layer chromatography (TLC), visualized by UV irradiation. The melting point (m.p.) of the compounds was determined using the open capillary method. The IR spectra were recorded with a Perkin Elmer FT-IR spectrometer in the frequency range 4000–400 cm^−1^. The ^1^H-NMR and ^13^C-NMR spectra were recorded on a Bruker AC (300 MHz for ^1^H-NMR) spectrometer using tetramethylsilane (TMS) as an internal standard in dimethyl sulfoxide (DMSO)-d_6_ for HAMQ and metal complexes (**C_1_**–**C_4_**). Chemical shifts (*δ*) are expressed in ppm. Mass spectra were recorded on a Waters SYNAPT G2 mass spectrometer using electrospray ionization (ESI-TOF) operating at an ionization potential of 70 eV. Ultraviolet and visible spectra of Schiff base and its complexes were recorded on a Shimadzu UV-3600 electronic spectrometer using quartz cells at room temperature in the range 200–1000 nm. Thermogravimetric analysis (TGA) and differential TGA (DTA) curves were generated in nitrogen atmosphere with a heating rate of 10 °C·min^−1^, in the temperature range 25–900 °C using platinum crucibles. Elemental analysis was calculated on a Thermo Finnigan Flash EA 1112 CHN analyzer. Molar conductivity data were recorded on a conductivity meter Model No. EQ-660A. X-ray powder analysis was obtained using an X-ray diffractometer (Rigaku Model-Mini Flex II) with a monochromatized Cu Kα X-ray beam of wavelength 0.15418 nm operated at 30 kV and 15 mA. The reagents required to perform the in vitro anticancer sulforhodamine B (SRB) assay such as Roswell Park Memorial Institute (RPMI)-1640, minimum essential medium (MEM), 10% fetal bovine serum, trypsin, trypan blue, ethanol, penicillin, streptomycin (0.5 mg/mL), dimethyl sulfoxide (DMSO), and sulforhodamine were procured from Sigma and Hi Media Ltd. India. Furthermore, a stock solution of 100 µg/mL for all test compounds was prepared by dissolving in 0.1% DMSO. The compounds were serially diluted and administered to the cancer cells. For the cell culture, we grew the cells and maintained them in an appropriate medium at pH 7.4, supplemented with 10% fetal bovine serum (FBS), glutamate (2 mM), penicillin, and streptomycin (0.5 mg/mL). The cell cultures used in this study were grown in a carbon dioxide incubator (Heraeus, GmbH, Germany) at 37 °C with 90% humidity and 5% CO_2_.

### 3.2. Computational Details

Following the methodology of our recent research [25,26,27,28,29,30,31,32,39,40], the computational determinations of the present study were done by resorting to the well-known Gaussian 09 software [33]. By considering the information acquired from those previous calculations, the MN12SX density functional [34] was selected due to the fact that it can be regarded as well behaved according to the “Koopmans in DFT” or KID assumption. Thus, the determination of the electronic properties was based on a model chemistry that relies on that density functional associated with the Def2TZVP basis set [41,42] for the estimation of the most stable structures. All calculations were done by considering DMSO as the solvent with the aid of the solvation model density (SMD) model [43] in order to get more accurate results. For the determination of the UV–Vis spectra, the same basis set and solvent were considered, but the density functional was replaced by the recommended CAM-B3LYP [44] considering 20 excited states.

### 3.3. Chemical Synthesis

#### 3.3.1. Synthesis of 3-[[(*E*)-(3-Hydroxyphenyl)methylidene]amino]-2-methyl- quinazolin-4(3*H*)-one (HAMQ)

The general synthetic route is showed in Scheme 1. For the synthesis of HAMQ, 0.122 g (1 mmol) of 3-hydroxybenzaldehyde was dissolved in 25 mL of dry ethanol. Then, 2–3 drops of glacial acetic acid were added as a catalyst during the reaction. To this clear solution, 0.175 g (1 mmol) of 2-amino-3-methyl-4(3*H*)-quinazolinone was added, which resulted in a transparent solution. Then, the reaction mixture was refluxed at 70 °C for 2 h. The pale-yellow precipitate obtained was filtered and washed several times with ethanol to obtain the final pure product. The purity of the products was checked by TLC.

Yield: 76%: m.p.: 258 °C. UV–Vis (DMSO, λ_max_): 298, 301, and 307 nm. ^1^H-NMR (DMSO-*d*_6_, 300 MHz): δ 8.633 (s, 1H, –N=CH–), δ 6.842–8.617 (m, 8H, Ar-H), δ 10.008 (s, 1H, Ar–OH), δ 2.588 (s, 3H, –CH_3_). FT-IR (KBr Pellet, cm^−1^) 3005.33 (–OH), 1669.55 (C=O), 1601.99 (C=N), 1431.94 (C=C), 1336.74, 1261.43 (C–O). LC–MS calculated for C_16_H_13_N_3_O_2_ [M]^+^: 280.29; found 281.1119 [M + H]. ^13^C-NMR [100 MHz, DMSO-d_6_, δ(ppm)]: 164.57 (–C=N–), 22.89 (CH_3_), 122.47–146.39 (Ar–C)

#### 3.3.2. Synthesis of Metal Complexes (**C_1_**–**C_4_**)

To an ethanolic solution (25 mL) of appropriate metal salts (1 mmol CdCl_2_·2H_2_O (0.219 g), NiCl_2_·6H_2_O, (0.237 g), ZnCl_2_·2H_2_O (0.154 g), or CuCl_2_·2H_2_O (0.170 g)), a solution of HAMQ in ethanol (25 mL) (1 mmol, 0.279 g) was added, followed by the addition of 1,10-phenanthroline (1 mmol, 0.198 g) to obtain a 1:1:1 (M:L:1,10-Phen) ratio. The resultant mixture was stirred under reflux for 6 h at 65 °C. The product obtained was filtered, washed off with ethanol, and dried under vacuum over CaCl_2_. The molecular formulas of the obtained complexes were as follows: **C_1_**: C_28_H_21_N_5_O_2_Cl_2_Cd; **C_2_**: C_28_H_21_N_5_O_2_Cl_2_Ni; **C_3_**: C_28_H_21_N_5_O_2_Cl_2_Cu; **C_4_**: C_28_H_21_N_5_O_2_Cl_2_Zn.

#### 3.3.3. In Vitro Antiproliferative Activity against Human Cancer Cell Lines

The in vitro cytotoxicity assay of the complexes was determined using the sulforhodamine B (SRB) assay. Briefly, the human cancer cell lines (HeLa and HCT-115) were grown at 37 °C in an atmosphere of 5% CO_2_ and 90% relative humidity in growth medium with FBS. Upon confluence, the monolayer cells were harvested with trypsin/ethylenediaminetetraacetic acid (EDTA) during the logarithmic growth phase, and the concentration of the suspension in cells/mL was counted using a hemocytometer. The cell density was adjusted to 1 × 10^4^ cells/well in the cell suspension. The plates were incubated at 37 °C in an atmosphere of 5% CO_2_ and 90% relative humidity for 24 h. After 24 h, the cells were washed with phosphate-buffered saline (PBS), and 50 μL of a serially diluted working solution of each test compound was separately added to a 96-well plate. The stock solutions of the test compounds (100 µg/mL), weighed as *w*/*v*, were prepared in DMSO and serially diluted with complete growth medium such that 100 μL of the working solution for each synthetic compound gave concentrations of 0.5, 0.25, 0.125, 0.0625, 0.0312, 0.0156, 0.0078, 0.0039, 0.0019, and 0.00097 μg/mL (final DMSO concentration was 0.5%–0.001%, respectively) added to the 96-well cell culture plates. An appropriate experimental blank, positive control, and vehicle blank were included in the assay containing the same concentration of DMSO. The standard drug doxorubicin was used at the concentration of 1 μg/mL (half maximal inhibitory concentration (IC_50_) 18.39 μM).

For the SRB assay, 50 μL of chilled 1% trichloroacetic acid (TCA) was slowly added to each well of the plates, obtaining a final concentration of 10%. The plates were incubated at 4 °C for 1 h to fix the cells to the bottom of the wells. The plates were then washed four times with distilled water and air-dried. To each well, 100 μL of SRB dye (0.4% *w*/*v* in 1% acetic acid) was added and left at room temperature for 60 min. The plates were then washed with 1% acetic acid to remove any unbound dye. The plates were again air-dried, and 100 μL of Tris buffer (10 mM; pH 10.5) was added to each well. The plates were shaken gently for 15 min on a mechanical shaker. Blank wells contained medium but no cells, and the control wells contained cells but no test samples. The optical density (OD) of the plate wells was recorded with a microplate reader at 510 nm. Percentage growth inhibition was calculated as follows:

(2) $$\%\;\mathrm{Growth}\;\mathrm{inhibition}=\frac{\left[\mathrm{OD}\;\mathrm{of}\;\mathrm{test}\;\mathrm{sample}-\mathrm{OD}\;\mathrm{of}\;\mathrm{blank}\right]}{\mathrm{OD}\;\mathrm{of}\;\mathrm{control}-\mathrm{OD}\;\mathrm{of}\;\mathrm{blank}}\times 100$$

## 4. Conclusions

In summary, a quinolin-4(3*H*)-one-based Schiff base ligand, 3-[(*E*)-(3-hydroxyphenyl)methylidene]amino-2-methylquinazolin-4(3*H*)-one (HAMQ), and its Cd(II), Ni(II), Cu(II), and Zn(II) complexes were synthesized and characterized using physicochemical techniques. The electronic absorption of the ligand and complexes was investigated in DMSO solution, which revealed that the bands shifted to longer wavelengths in the complexes due to the coordination of the ligand with the metal ions. The results of in vitro anticancer activity determined using the SRB assay demonstrated that the synthesized complexes exhibited progressive anticancer activity against human cervical cancer HeLa cells and human colon cancer HCT-115 cells, suggesting their potential use as anticancer agents, subject to further careful investigations including small animal model experiments, as well as pharmacokinetic and pharmacodynamics studies. Furthermore, the MN12SX/Def2TZVP/DMSO model chemistry was validated through a fulfillment of the KID methodology allowing the direct determination of the chemical reactivity descriptors from the results of the HOMO and LUMO energy values of the ground state of the studied compounds. The simulated UV–visible spectrum, determined with the aid of the CAM-B3LYP density functional basis set and the TD-DFT technique, was comparable with the experimental results. From the calculation of the global reactivity descriptors, three conclusions arose, giving information related to the electron-donating characteristic of the studied compounds, as well as the differences in chemical reactivity based on the values of global hardness, with varying influence of the complexing metals on the reactivity properties.

## Figures and Tables

**Table 1:** Unit cell parameters, volume, and crystallite size of metal complexes. FoM, figure of merit.

Compound	Lattice Constants (A˚)	Angles (˚)	FoM	Volume (Å)	Crystallite Size (Å)
**C_1_**	a = 4.8 b = 14.1 c = 15.8	α = 55.11 β = 82.97 ϓ = 41.62	1.89	1069.34	570.62
**C_2_**	a = 5.3 b = 7.4 c = 18.4	α = 56.55 β = 85.65 ϓ = 90.48	9.95	721.65	432.81
**C_3_**	a = 4.9 b = 9.4 c = 23.3	α = 48.20 β = 61 ϓ = 58.99	3.76	1073.20	312.51
**C_4_**	a = 2.5 b = 7.3 c = 13.7	α = 50.80 β = 67.40 ϓ = 69.06	2.23	250.03	1336.94

**Table 2:** Concentrations of the complexes used in SRB assay with the values converted to µM. M_W_, molecular weight.

Concentration (µg/mL)	IC_50_ (µM)				
	**C_1_** (M_W_ 643)	**C_2_** (M_W_ 587)	**C_3_** (M_W_ 631)	**C_4_** (M_W_ 593)	HAMQ (M_W_ 302)
0.5	77.26	36.58	35.64	50.17	16.56
0.250	74.78	35.11	31.65	42.98	8.28
0.125	73.83	27.8	29.9	31.86	4.14
0.063	67.12	14.69	28.64	30.5	2.07
0.031	59.49	9.23	21.3	22.73	1.03
0.016	49.91	7.31	17.03	22.38	0.52
0.008	24.72	3.88	15.59	22.17	0.26
0.004	150.1	−0.62	12.34	8.42	0.13
0.002	13.43	0.27	10.42	4.05	0.06
0.001	7.43	−2.69	8.14	2.86	0.03
Doxorubicin	18.40				

**Table 3:** The orbital energies of the highest occupied, lowest unoccupied, and singly occupied molecular orbitals (HOMO, LUMO, and SOMO), as well as the HOMO–LUMO (H–L) gap (all in eV) of the **HAMQ** ligand and its four metallic complexes; the $J_{I}$, $J_{A}$, $J_{HL}$ and ΔSL descriptors were estimated with the aid of MN12SX/Def2TZVP/DMSO model chemistry.

Compound	HOMO	LUMO	SOMO	H–L gap	$J_{I}$	$J_{A}$	$J_{HL}$	ΔSL
HAMQ	−6.16	−2.21	−2.25	3.95	0.01	0.02	0.02	0.04
**C_1_**	−6.08	−2.57	−2.57	3.51	0.06	0.00	0.06	0.00
**C_2_**	−6.16	−2.62	−2.61	3.54	0.02	0.00	0.02	0.01
**C_3_**	−5.89	−2.69	−2.61	3.28	0.02	0.01	0.06	0.08
**C_4_**	−6.05	−2.69	−2.67	3.36	0.06	0.01	0.06	0.02

**Table 4:** Global reactivity descriptors (in eV) of the **HAMQ** ligand and its four metallic complexes calculated with the aid of the MN12SX/Def2TZVP/DMSO model chemistry.

Compound	χ	η	ω	$\omega^{-}\;$	$\omega^{+}\;$	$\Delta \omega^{\pm }$
HAMQ	4.19	3.95	2.22	6.78	2.59	9.37
**C_1_**	4.32	3.52	2.66	7.70	3.38	11.08
**C_2_**	4.39	3.54	2.72	7.85	3.46	11.31
**C_3_**	4.29	3.20	2.87	8.08	3.79	11.88
**C_4_**	4.39	3.54	2.72	7.85	3.46	11.31

## Supplementary Materials

The following are available online, Figure S1: ^1^H-NMR spectrum of **HMAQ** ligand, Figure S2: ^1^H-NMR spectrum of **C_1_**, Figure S3: ^1^H-NMR spectrum of **C_4_**, Figure S4: ^13^C-NMR spectrum of **HAMQ**, Figure S5: ^13^C-NMR spectrum of **C_1_**, Figure S6: FT-IR spectrum of **HAMQ** ligand, Figure S7: FT-IR spectrum of **C_1_**, Figure S8: FT-IR spectrum of **C_4_**, Figure S9: Graphical sketches of the simulated IR spectra of (a) **HAMQ**, (b) **C_1_**, (c) **C_2_**, (d) **C_3_** and (e) **C_4_** complexes, Figure S10: UV-Visible spectra of **HAMQ** and its complexes in DMSO solution, Figure S11: Graphical sketches of the UV-Visible spectra of (a) **HAMQ**, (b) **C_1_**, (c) **C_2_**, (d) **C_3_** and (e) **C_4_** complexes, Figure S12: Mass spectrum of **HAMQ** ligand, Figure S13: Mass spectrum of **C_1_**, Figure S14: Mass spectrum of **C_2_**, Figure S15: Mass spectrum of **C_3_**, Figure S16: Mass spectrum of **C_4_**, Figure S17: TGA/DTG curves of **C_1_**–**C_4_** complexes, Figure S18: XRD pattern of **C_1_**–**C_4_** complexes, Table S1: Analytical and physical data of the metal complexes, Table S2: Important UV-Vis spectral bands of **HAMQ** ligand and its metal complexes, Table S3: Stepwise thermal degradation of metal complexes, Table S4: Crystallographic data of complex **C_1_**, Table S5: Crystallographic data of complex **C_2_**, Table S6: Crystallographic data of complex **C_3_**, Table S7: Crystallographic data of complex **C_4_**.
