# Supplementary material for: Preparation, Spectroscopic Characterization, Theoretical Investigations, and In Vitro Anticancer Activity of Cd(II), Ni(II), Zn(II), and Cu(II) Complexes of 4(3H)-Quinazolinone-Derived Schiff Base
Source: Molecules. 2020 Dec 16;25(24):5973. doi: 10.3390/molecules25245973 (PMC7766817; doi:10.3390/molecules25245973)
Supplement: Supplementary file 1 [file molecules-25-05973-s001.pdf]

Supporting Information

Preparation, Spectroscopic Characterization, Theoretical Investigations and *In Vitro* Anticancer Activity of Cd(II), Ni(II), Zn(II) and Cu(II) Complexes of 4(3*H*)-Quinazolinone Derived Schiff Base

Ubale Panchsheela Ashok [1, 2], Shiva Prasad Kollur [3, *], Nishad Anil [4], Bansode Prakash Arun [5], Jadhav Sanjay Namdev [6], Sanjay Sarasamkar [7], Helavi Vasant Baburao [1, *], Asha Srinivasan [8], Sandeep Kaulage [9], Ravindra Veerapur [10], Sarah Al-Rashed [11], Asad Syed [11], Joaquín Ortega Castro [12], Juan Frau [12], Norma Flores-Holguín [13], Daniel Glossman-Mitnik [12, 13]

[1] Department of Chemistry, Rajaram College, Kolhapur - 416 004, Maharashtra, India. panchsheela_ubale@rediffmail.com (U.P.A.)
[2] N.K. Orchid college of Engineering and Technology, Solapur - 413 002, Maharashtra, India.
[3] Department of Sciences, Amrita School of Arts and Sciences, Amrita Vishwa Vidyapeetham, Mysuru campus, Mysuru - 570 026, Karnataka, India.
[4] Department of Chemistry, Institute of Science, Mumbai - 400 032, Maharashtra, India. anilnishad789@gmail.com (N.A.)
[5] Department of Chemistry, Sangola College Sangola, Sangola - 413 307, Maharashtra, India. bansode.prakash4@gmail.com (B.P.A.)
[6] Organic Chemistry Division, CSIR-National Chemical Laboratory, Pune – 411 008, Maharashtra, India. sanjay.jadhav31@gmail.com (S.N.)
[7] Department of Chemistry, Walchand Institute of Technology, Solapur - 413 002, Maharashtra, India. sarsamkar30@yahoo.co.in (S.S.)
[8] Division of Nanoscience and Technology, Faculty of Life Sciences, JSS Academy of Higher Education and Research, Mysuru – 570 015, Karnataka, India. asha.srinivasan@jssuni.edu.in (A.S.)
[9] Department of Chemistry, Indian Institute of Science, Education and Research, Pune – 411 008, Maharashtra, India. sandeep.kaulage@gmail.com (S.K.)

[10] Department of Materials and Metallurgy Engineering, Malawi Institute of Technology, Malawi University of Science and Technology, P.O . Box - 5916, Limbe, Malawi. rveerapur@must.ac.mw (R.V.)
[11] Department of Botany and Microbiology, College of Science, King Saud University, P.O. Box 2455, Riyadh 11451, Saudi Arabia. salrashed@ksu.edu.sa (S.A.R.); assyed@ksu.edu.sa (A.S.)
[12] Departament de Química, Universitat de les IllesBalears, Palma de Mallorca 07122, Spain. joaquin.castro@uib.es (J.O.C.); juan.frau@uib.es (J.F.)
[13] Laboratorio Virtual NANOCOSMOS, Departamento de Medio Ambiente y Energía, Centro de Investigaci´on en MaterialesAvanzados, Chihuahua, Chih 31136, Mexico. norma.flores@cimav.edu.mx (N.F.H.); daniel.glossman@cimav.edu.mx (D.G.M.)

*Correspondence: shivachemist@gmail.com (S.P.K.); vbhelavi@gmail.com (H.V.B.)

Contents:

**Figure S1:**	^{1}H-NMR spectrum of **HMAQ** ligand.
**Figure S2:**	^{1}H-NMR spectrum of C_1.
**Figure S3:**	^{1}H-NMR spectrum of C_4.
**Figure S4:**	^{13}C-NMR spectrum of **HAMQ.**
**Figure S5:**	^{13}C-NMR spectrum of C_1.
**Figure S6:**	FT-IR spectrum of **HAMQ** ligand.
**Figure S7:**	FT-IR spectrum of C_1.
**Figure S8:**	FT-IR spectrum of C_4.
**Figure S9:**	Graphical sketches of the simulated IR spectra of a) **HAMQ**, b) C_1, c) C_2, d) C_3 and e) C_4 complexes.
**Figure S10:**	UV-Visible spectra of **HAMQ** and its complexes in DMSO solution.
**Figure S11:**	Graphical sketches of the UV-Visible spectra of a) **HAMQ**, b) C_1, c) C_2, d) C_3 and e) C_4 complexes.
**Figure S12:**	Mass spectrum of **HAMQ** ligand.
**Figure S13:**	Mass spectrum of C_1.
**Figure S14:**	Mass spectrum of C_2.
**Figure S15:**	Mass spectrum of C_3.
**Figure S16:**	Mass spectrum of C_4.
**Figure S17:**	TGA/DTG curves of C_1-C_4 complexes.
**Figure S18:**	XRD pattern of C_1-C_4 complexes.
**Table S1:**	Analytical and physical data of the metal complexes.
**Table S2:**	Important UV-Vis spectral bands of **HAMQ** ligand and its metal complexes.
**Table S3:**	Stepwise thermal degradation of metal complexes
**Table S4:**	Crystallographic data of complex C_1.
**Table S5:**	Crystallographic data of complex C_2.
**Table S6:**	Crystallographic data of complex C_3.
**Table S7:**	Crystallographic data of complex C_4.
	**Expressions used to calculate global reactivity descriptors**

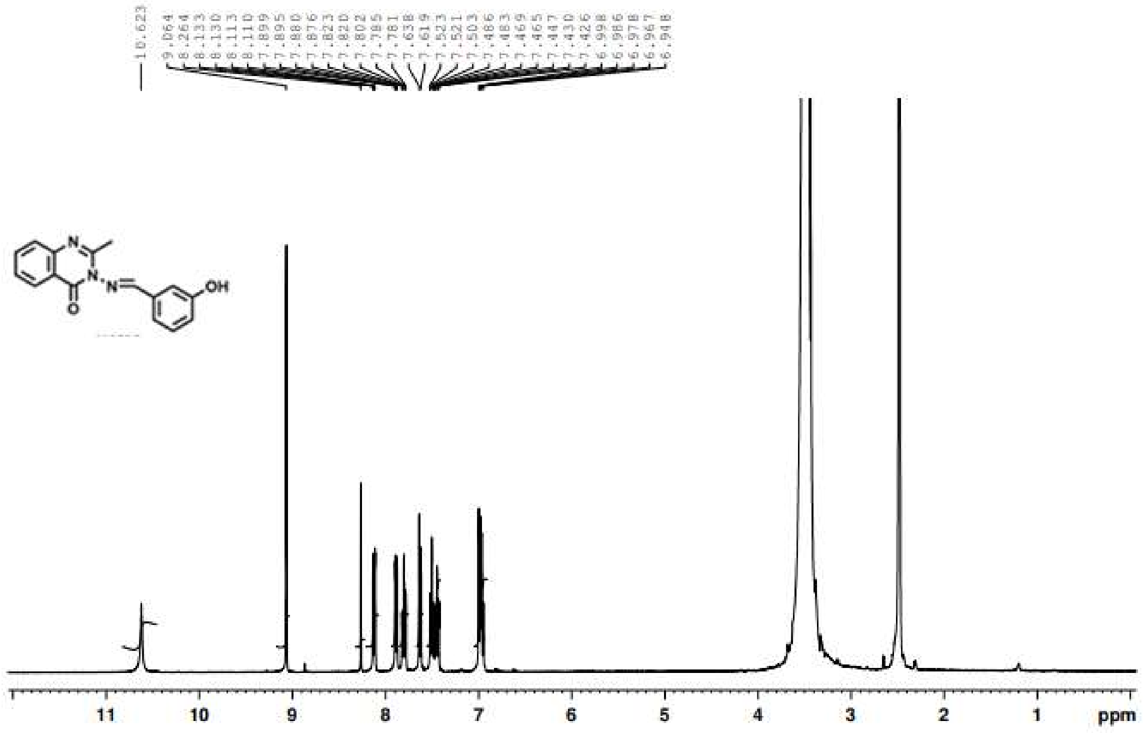

Figure S1: ^{1}H-NMR spectrum of **HMAQ** ligand.

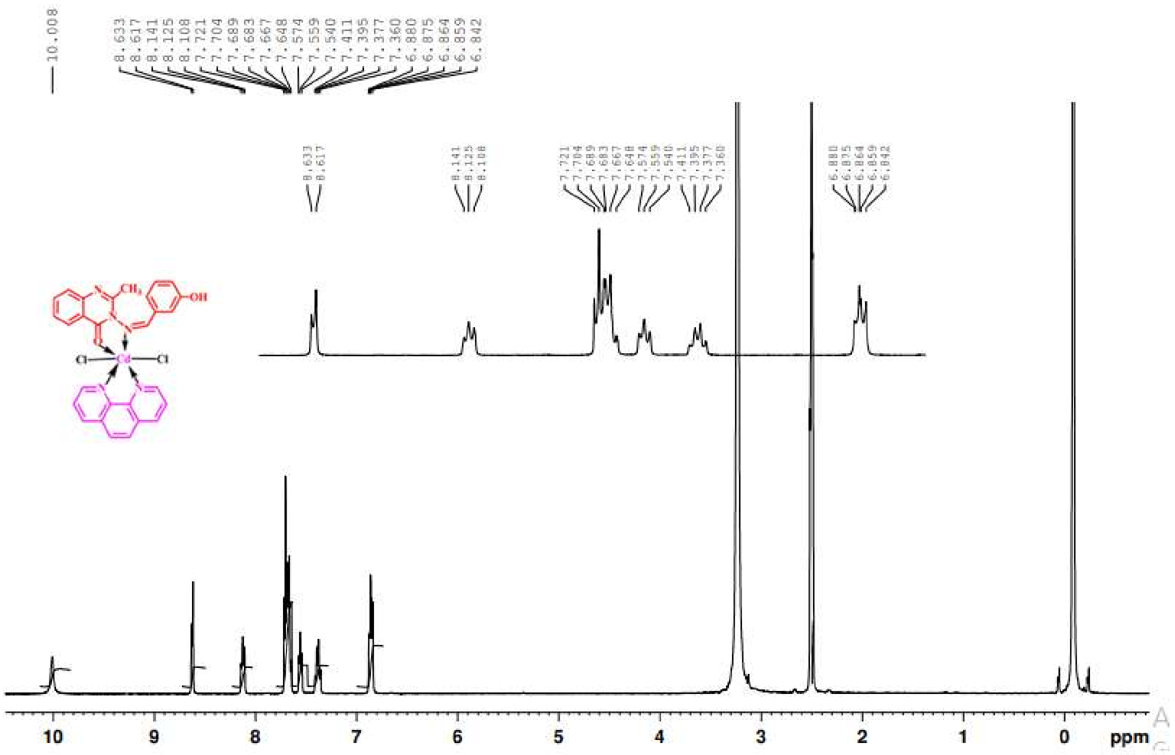

Figure S2: ^{1}H-NMR spectrum of C_1.

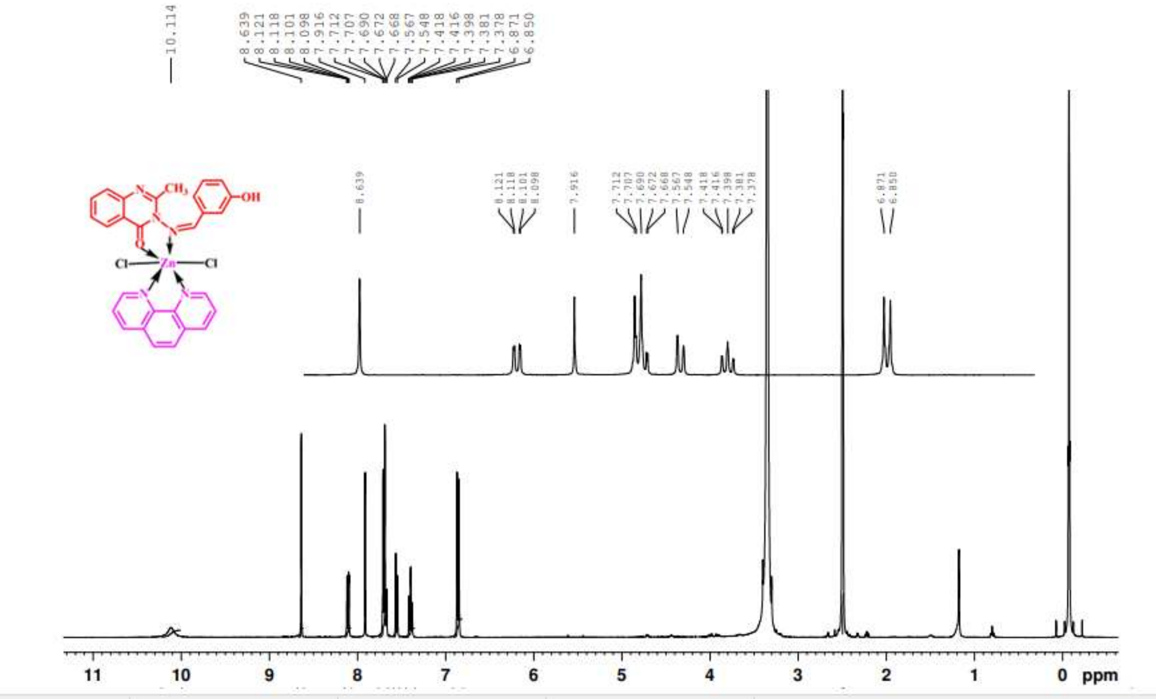

Figure S3: ^{1}H-NMR spectrum of C_3.

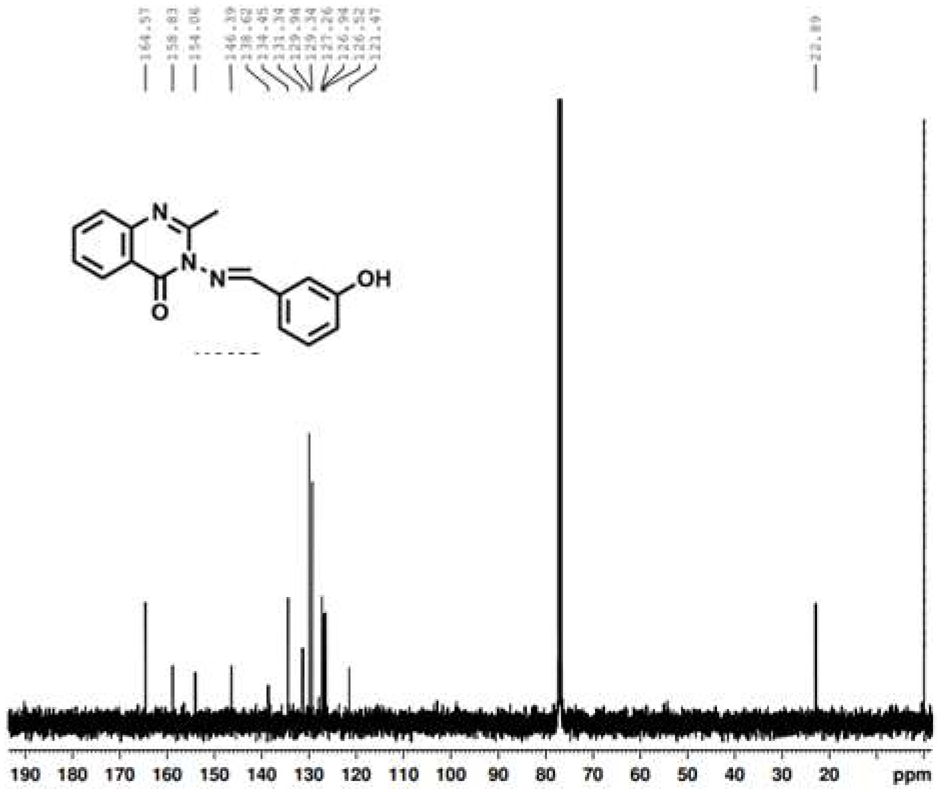

Figure S4: ^{13}C-NMR spectrum of **HAMQ**.

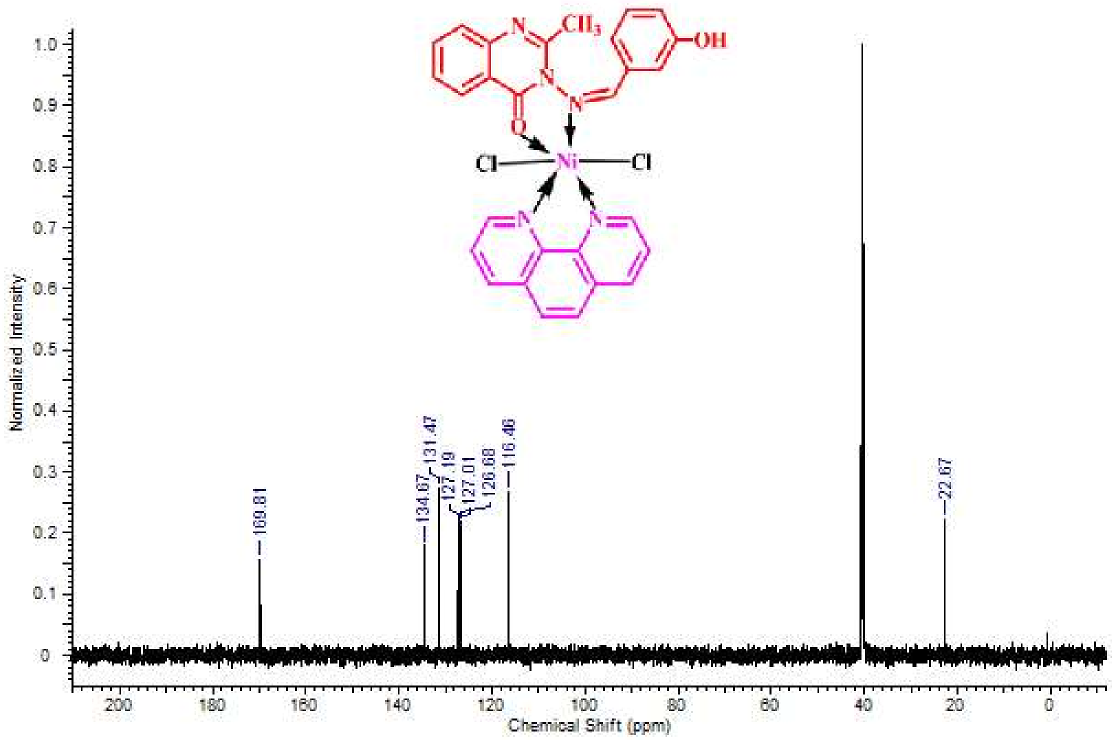

Figure S4: ^{13}C-NMR spectrum of **C_2**.

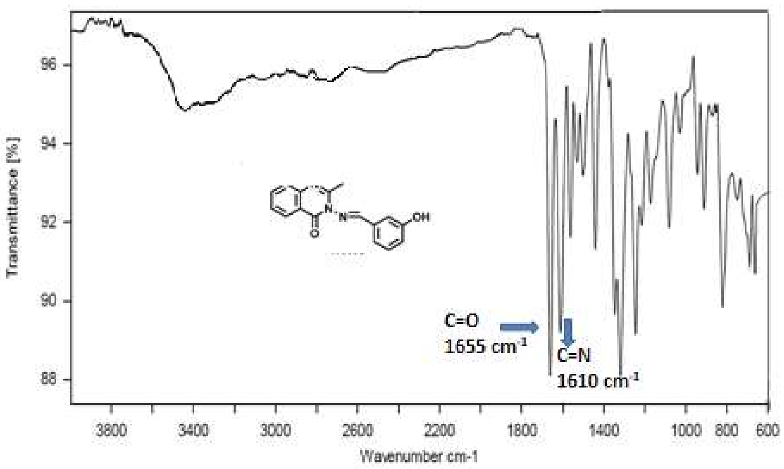

Figure S6: FT-IR spectrum of **HAMQ** ligand.

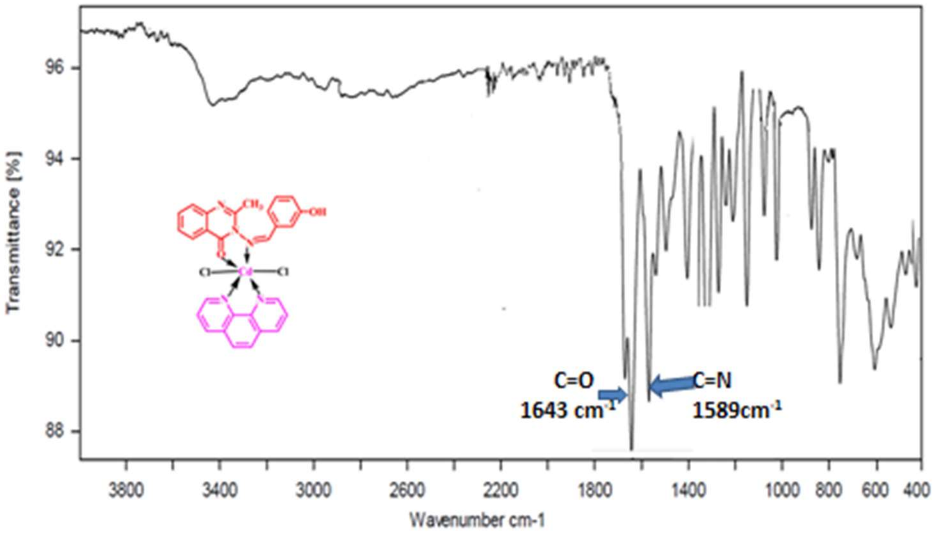

Figure S7: FT-IR spectrum of C_1.

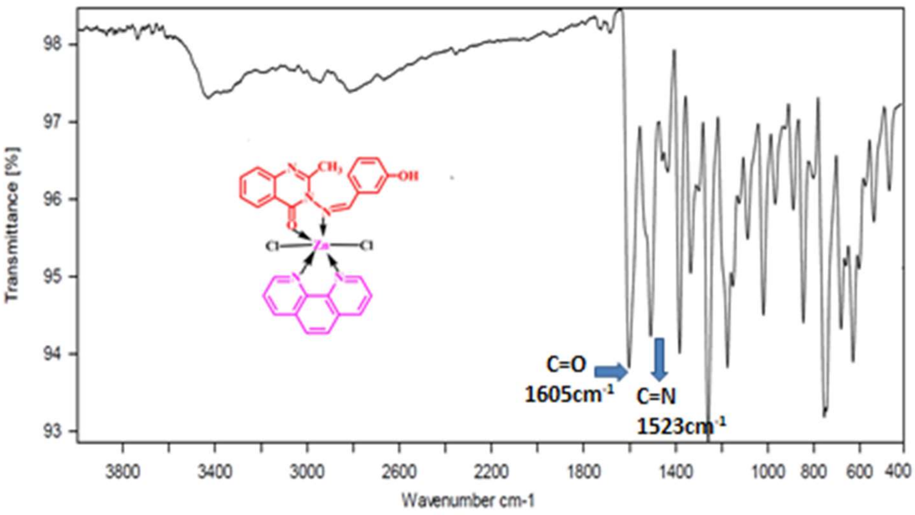

Figure S8: FT-IR spectrum of C_3.

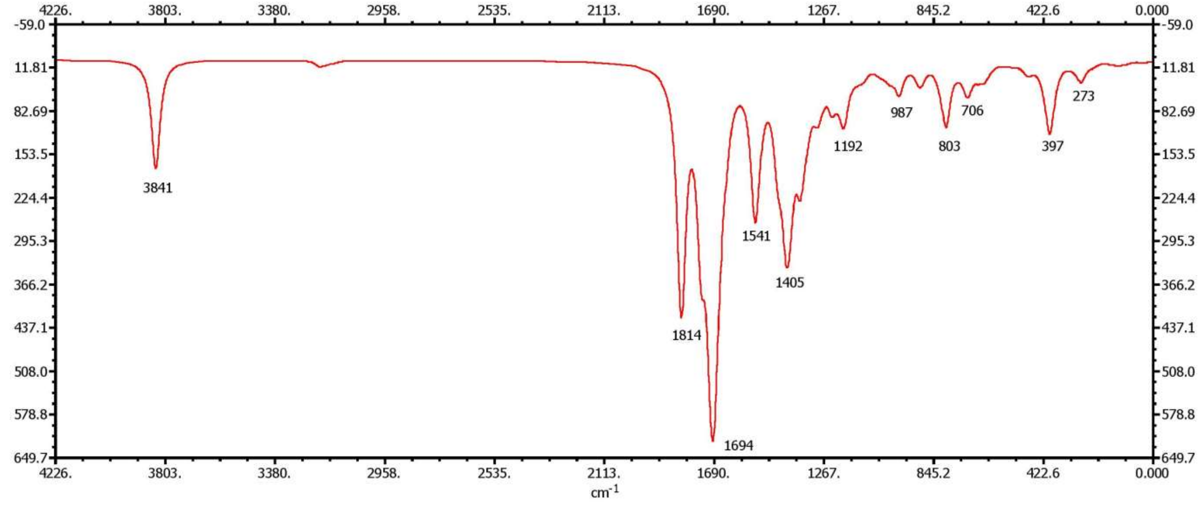
3841
1814
1694
1541
1405
1192
987
803
706
397
273
cm-1

(a)

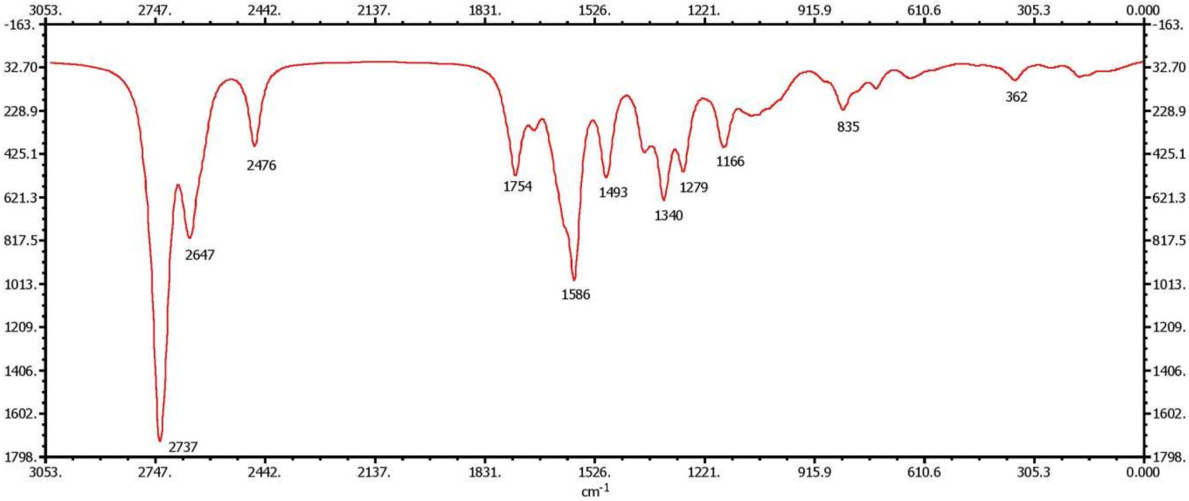
2737
2647
2476
1754
1586
1493
1340
1279
1166
835
362
cm-1

(b)

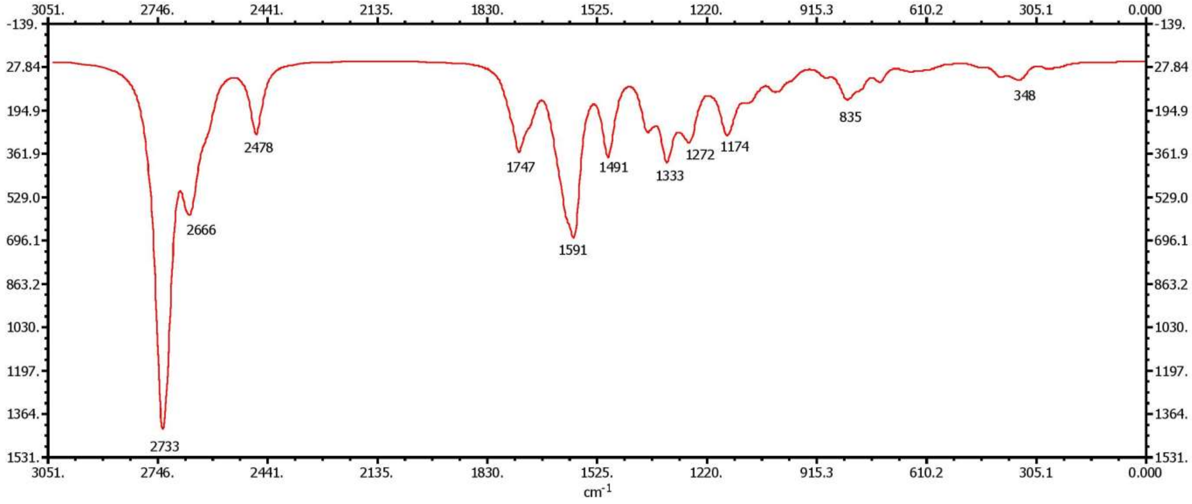

3051.
2746.
2441.
2135.
1830.
1525.
1220.
915.3
610.2
305.1
0.000
-139.
27.84
194.9
361.9
529.0
696.1
863.2
1030.
1197.
1364.
1531.
2733
2666
2478
1747
1591
1491
1333
1272
1174
835
348
cm-1

(c)

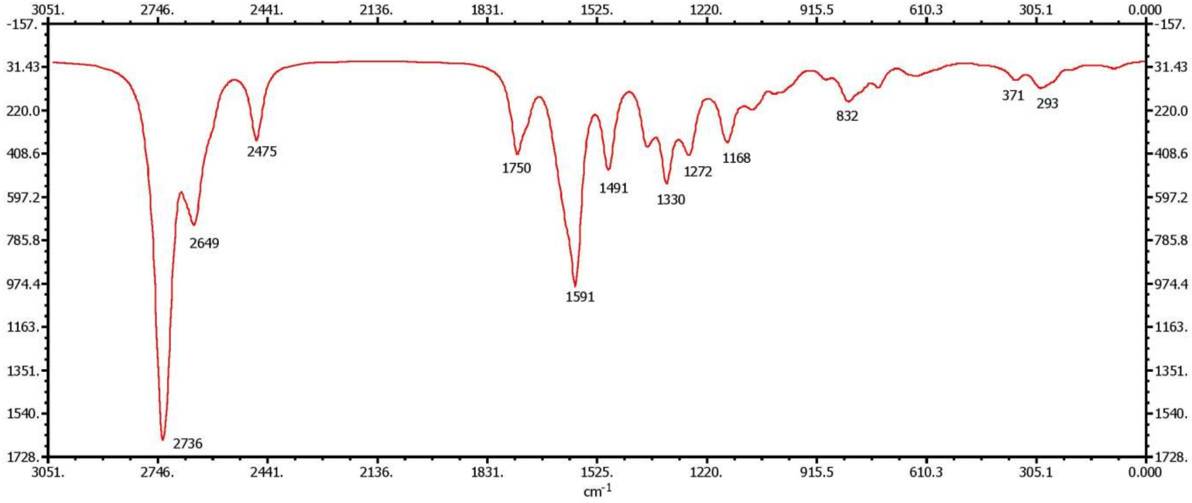

3051.
2746.
2441.
2136.
1831.
1525.
1220.
915.5
610.3
305.1
0.000
-157.
31.43
220.0
408.6
597.2
785.8
974.4
1163.
1351.
1540.
1728.
2736
2649
2475
1750
1591
1491
1330
1272
1168
832
371
293
cm-1

(d)

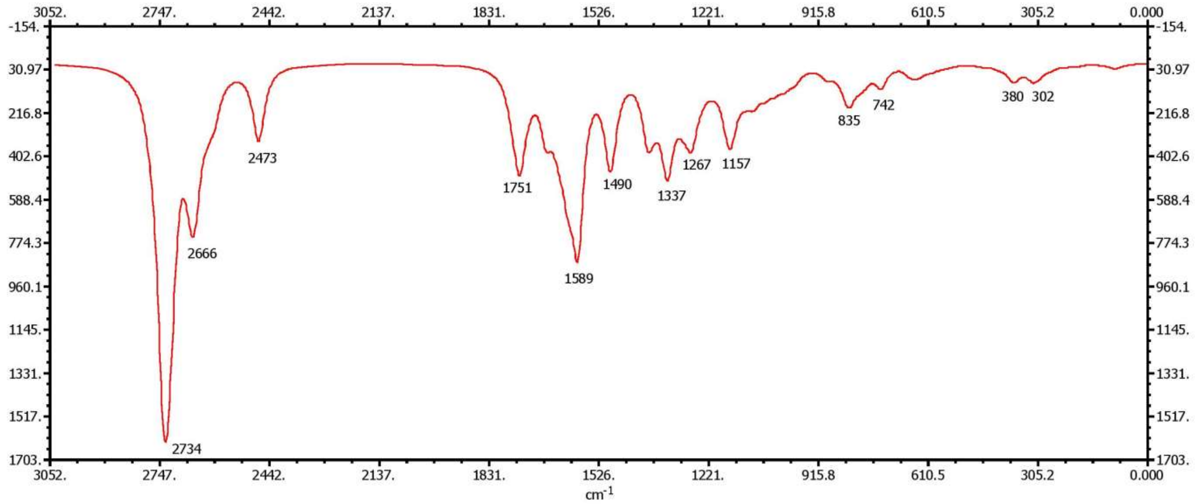

(e)

Figure S9: Graphical sketches of the simulated IR spectra of a) **HAMQ**, b) C_1, c) C_2, d) C_3 and e) C_4 complexes.

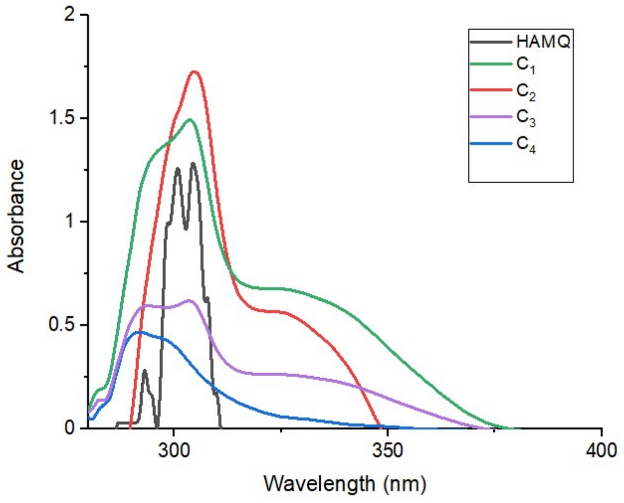

Figure S10: UV-Visible spectra of **HAMQ** and its complexes in DMSO solution.

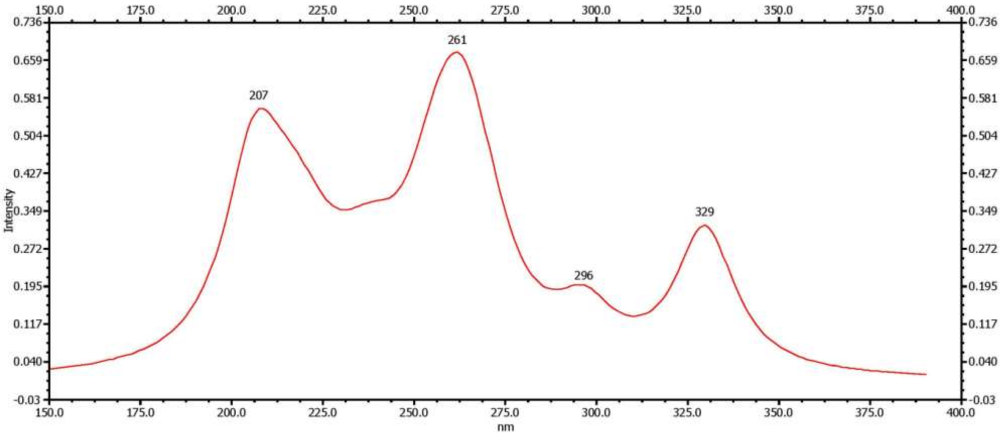
Intensity
nm
207
261
296
329

(a)

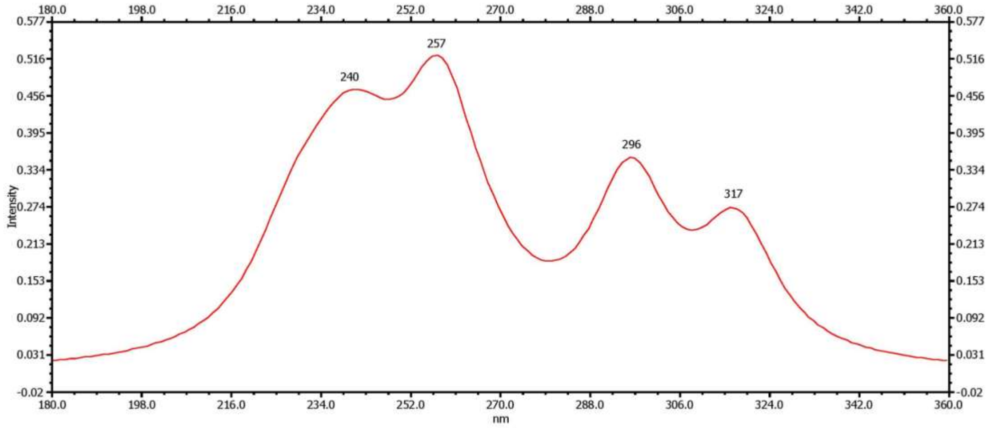
Intensity
nm
240
257
296
317

(b)

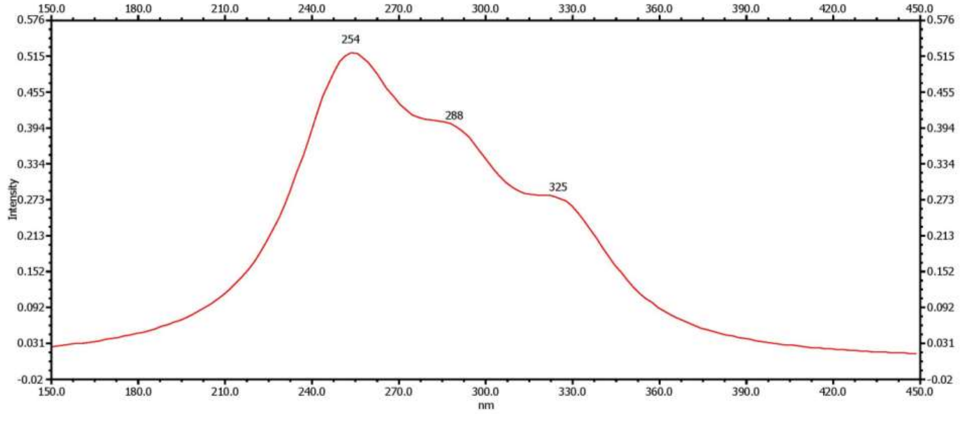
Intensity
nm
254
288
325

(c)

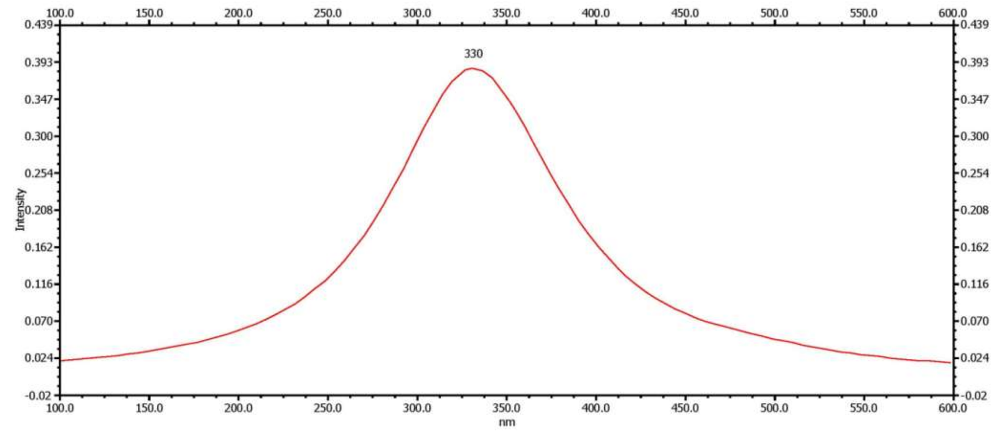

(d)

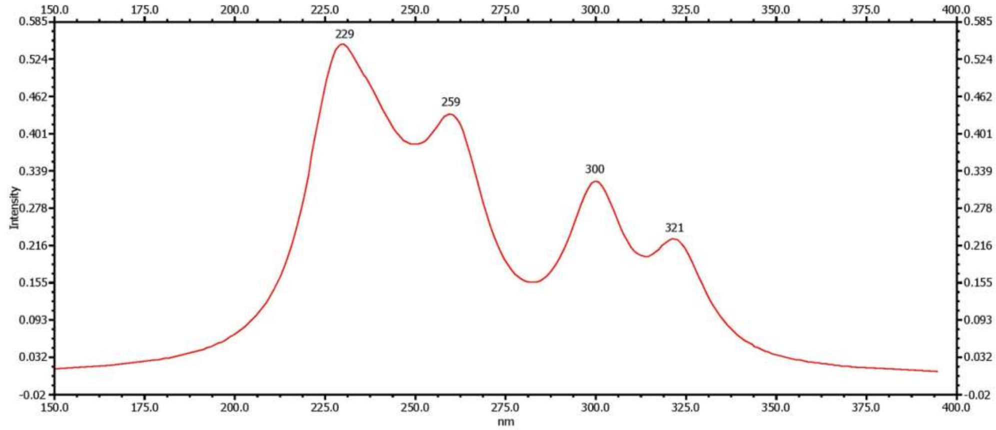

(e)

Figure S11: Graphical sketches of the UV-Visible spectra of a) **HAMQ**, b) C1, c) C2, d) C3 and e) C4 complexes.

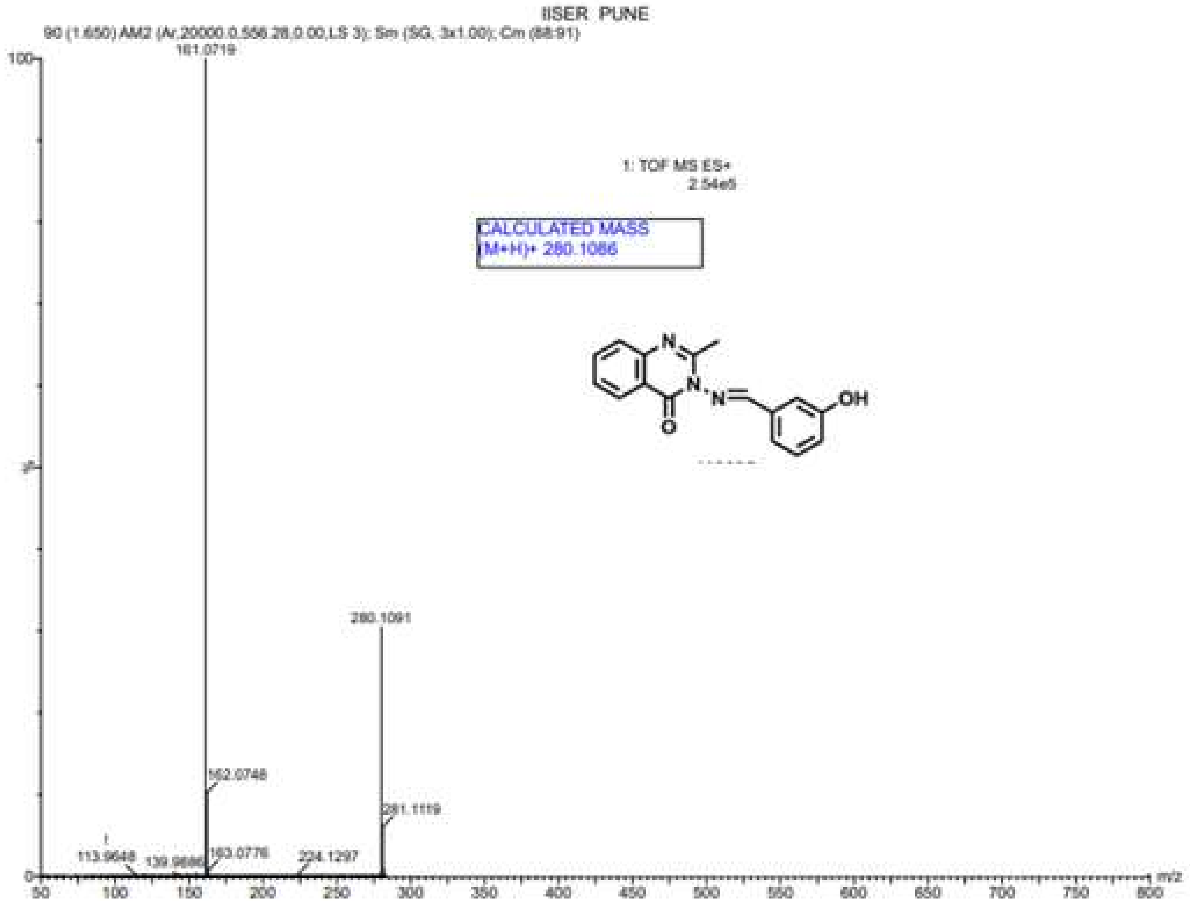

Figure S12: Mass spectrum of **HAMQ** ligand depicting $[M+H]^+$ at m/z = 281.1119.

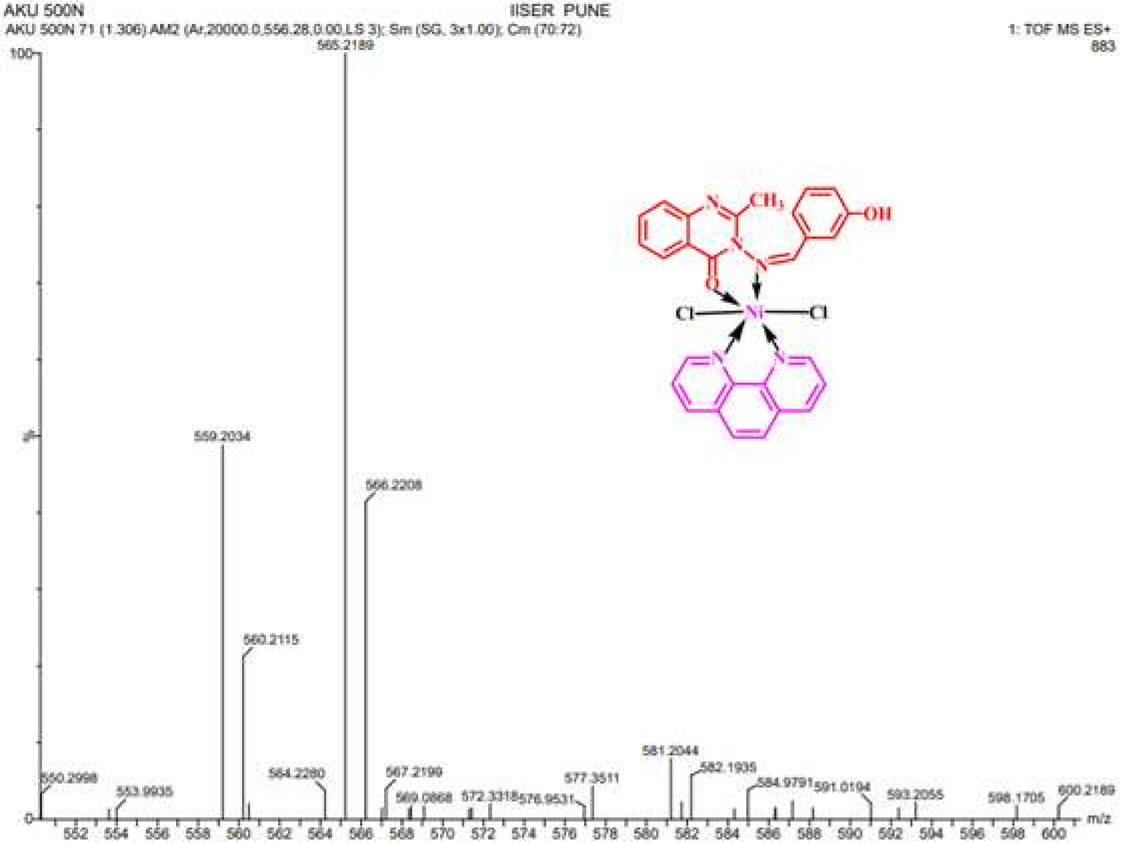

Figure S13: Mass spectrum of C_2 showing [M+2] at m/z = 591.0194.

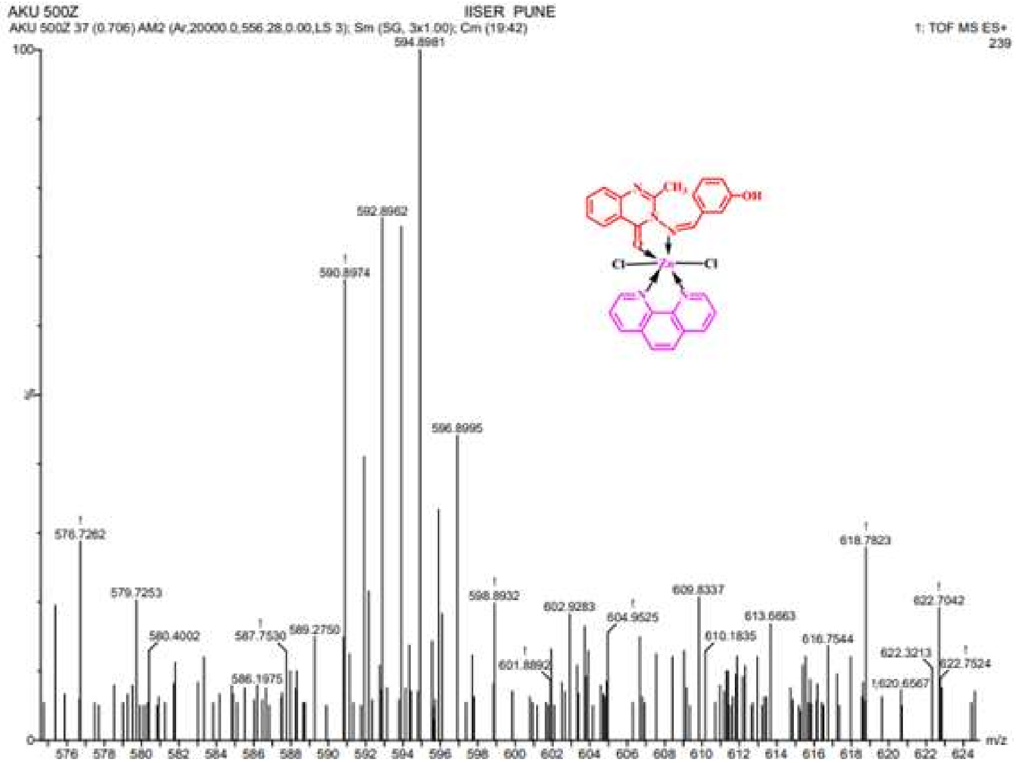

Figure S14: Mass spectrum of C3 revealing $[M]^+$ at *m*/*z* = 594.8981.

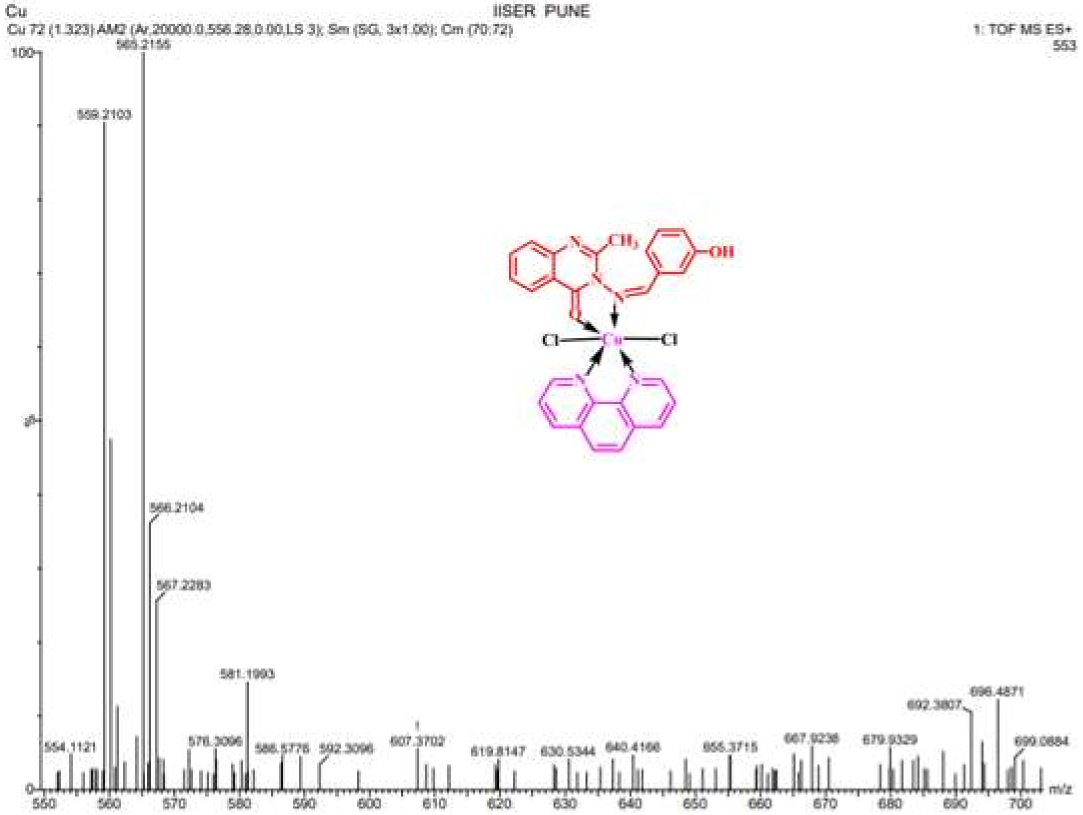

Figure S15: Mass spectrum of C_4 showing [M+2] at *m*/*z* = 592.3096.

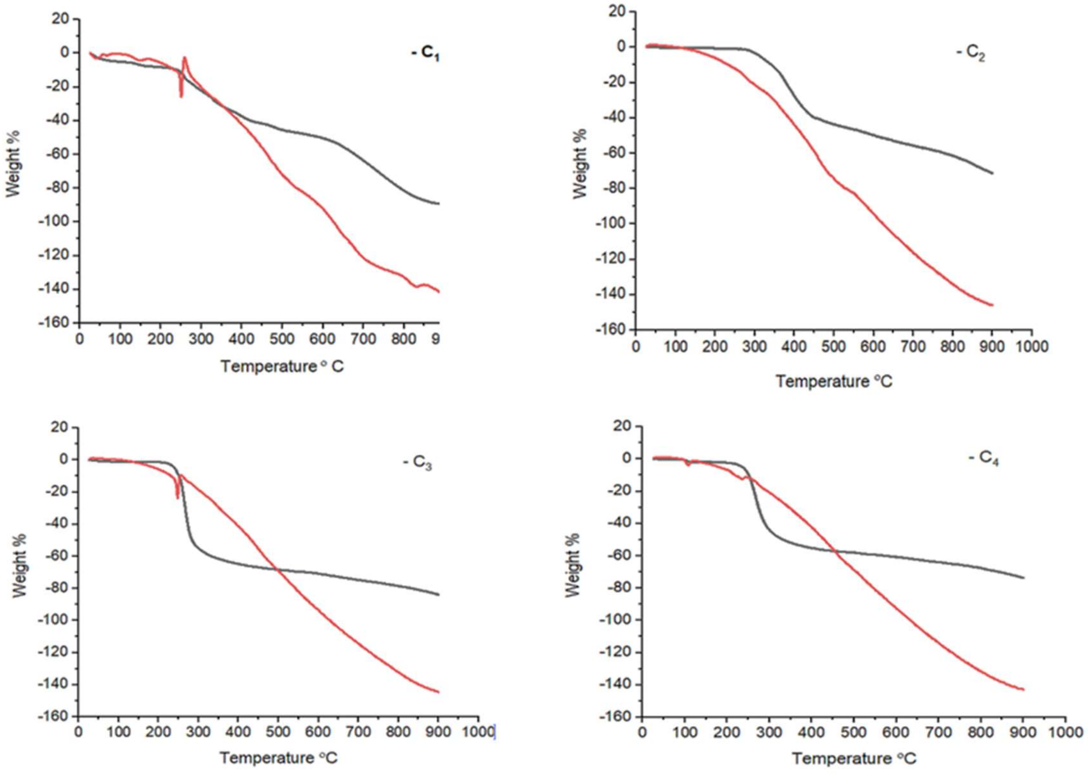

Figure S16: TGA/DTG curves of C_1-C_4 complexes.

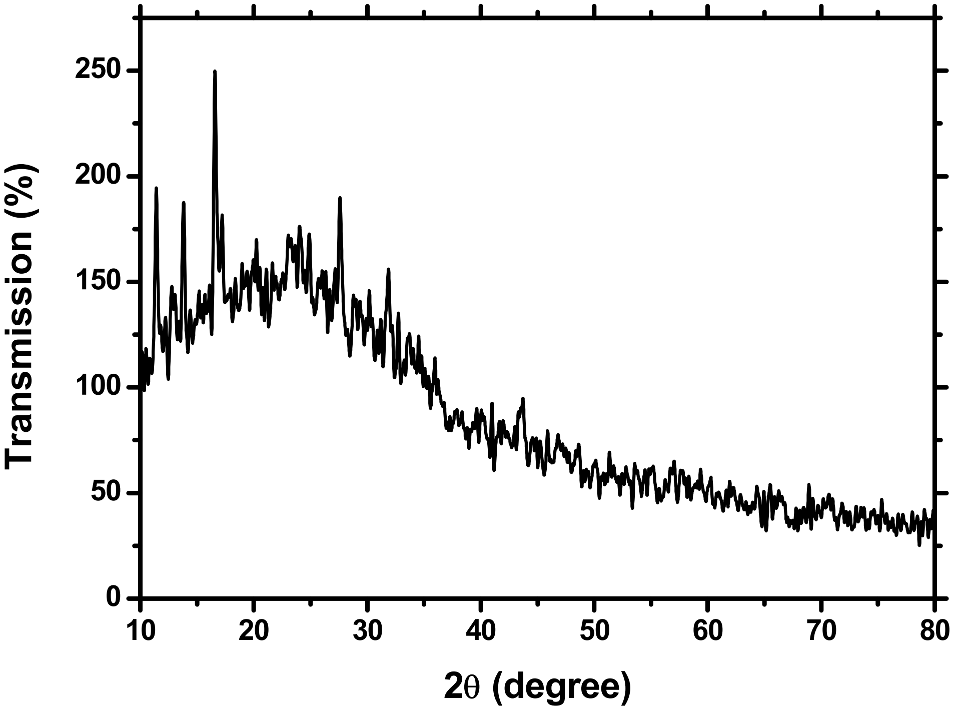
Transmission (%)
250
200
150
100
50
0
10
20
30
40
50
60
70
80
2θ (degree)

(a)

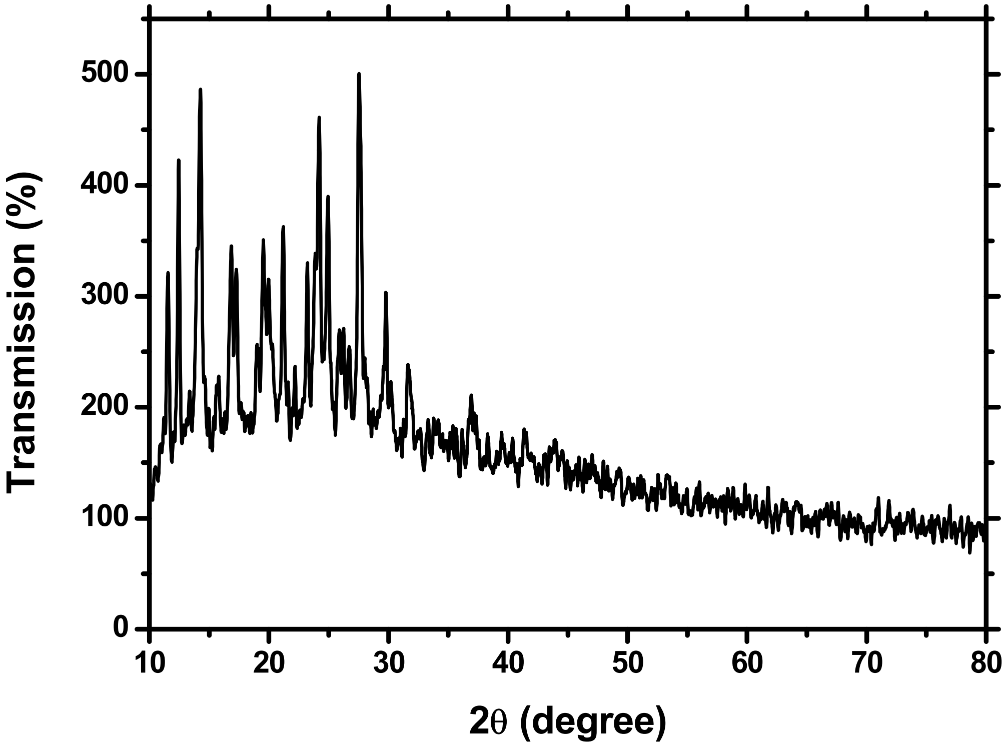
Transmission (%)
2θ (degree)
0
100
200
300
400
500
10
20
30
40
50
60
70
80

(b)

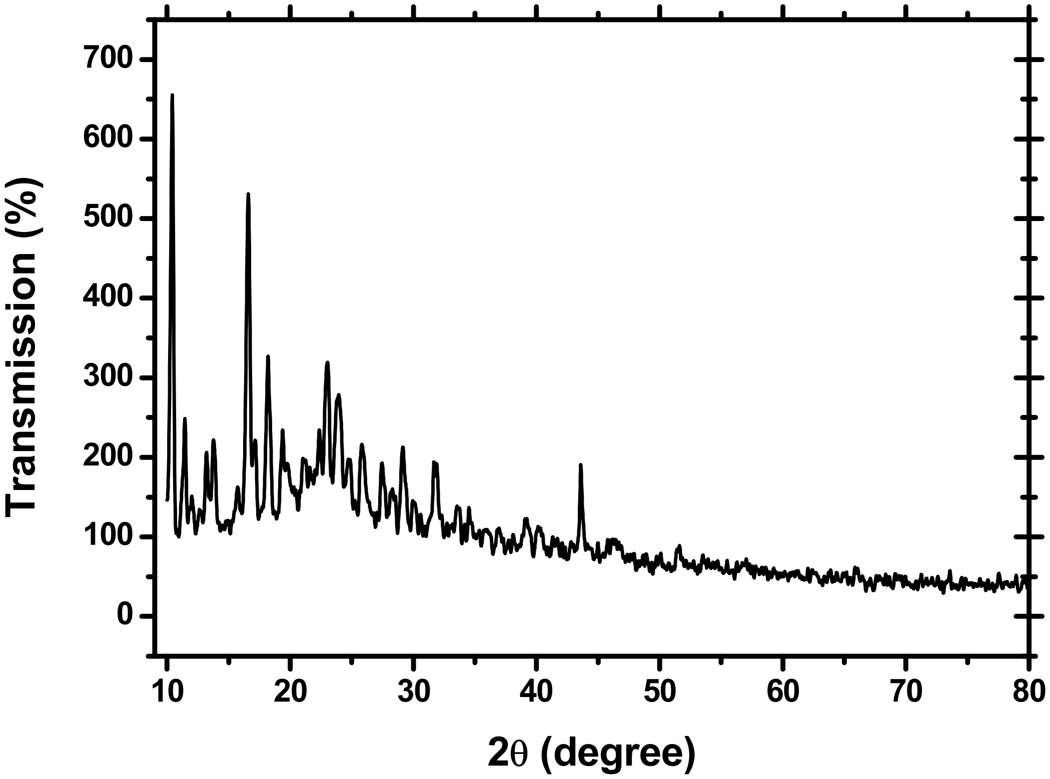
Transmission (%)
700
600
500
400
300
200
100
0
10
20
30
40
50
60
70
80
2θ (degree)

(c)

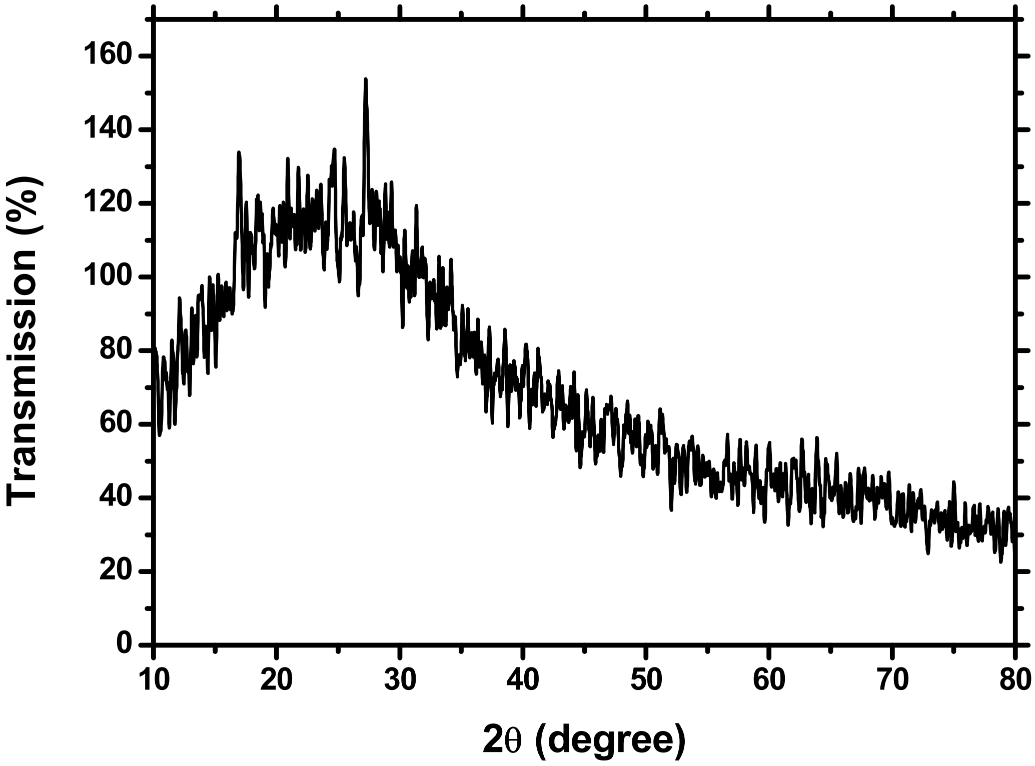

(d)

Figure S17: XRD pattern of C_1-C_4 complexes.

Table S1: Analytical and physical data of the metal complexes.

Compound	Molecular Formula	Yield (%)	(Calculated) Found %				Molecular Conductance (Ω^{-1} cm^2mol^{-1})
			C	H	N	M	
**C1**	$C_{28}H_{21}N_5O_2Cl_2$ Cd	63	(52.32) 52.19	(3.29) 3.66	(10.89) 10.74	(17.49) 17.60	24.08
**C2**	$C_{28}H_{21}N_5O_2Cl_2$ Ni	57	(57.84) 57.88	(3.84) 3.89	(12.82) 12.85	(9.84) 9.87	22.54
**C3**	$C_{28}H_{21}N_5O_2Cl_2$ Cu	64	(55.81) 55.93	(3.82) 3.91	(12.78) 12.97	(9.61) 9.72	17.93
**C4**	$C_{28}H_{21}N_5O_2Cl_2$ Zn	62	(56.44) 56.40	(3.56) 3.53	(11.75) 11.72	(10.98) 10.94	15.91

Table S2: Important IR spectral bands of HAMQ ligand and its metal complexes.

Compound	υ(-OH)	υ(C=O)	υ(HC=N)	υ(M-O)
HAMQ	3005	1669	1601	-
C1	3226	1623	1588	540
C2	3219	1650	1588	536
C3	3221	1620	1592	538
C4	3300	1621	1588	533

Table S3: Important UV-vis spectral bands of HAMQ ligand and its metal complexes.

**Compound**	**λ_{max} in nm**	**ε_{max} in M^{-1} cm^{-1}**
HAMQ	298, 301, 307	33557, 33222, 32573
C2	690	14493
C3	678,709	14749, 14085
C4	712	14045

Table S4: Stepwise thermal degradation of metal complexes.

Compound	Thermogravimetry (TG)		Mass loss (%)		Decomposition product loss
	Stage	Temp (^oC)	Found	Calculated	
C1	I<br>II	26-250<br>250-900	1.62<br>82.1	2.32<br>82.15	Cl<br>Organic Moiety
C2	I<br>II	26-220<br>220-430<br>430-596<br>596-900	8.72<br>32.12<br>7.34<br>41.08	8.37<br>33.69<br>7.81<br>39.98	Cl<br>1,10-phen<br>$-N_2$<br>Fraction of Ligand
C3	I<br>II	26-230<br>200-800	2.42<br>71.03	2.50<br>71.77	Cl<br>Organic Moiety
C4	I<br>II	26-380<br>380-815	56.46<br>15.17	58.75<br>15.44	Fraction of Ligand<br>Remaining moiety

Table S5: Crystallographic data of C_1.

Pos. [°2Th.]	Height [cts]	FWHM [°2Th.]	d-spacing [Å]	Rel. Int. [%]	h	k	l
9.6171	19.54	0.2362	9.19658	48.91	0	1	1
11.406	21.24	0.2362	7.75792	100.00	0	1	2
13.8182	17.58	0.2362	6.40861	6.01	0	1	0
16.5771	30.86	0.1574	5.34774	5.74	0	0	2
19.775	24.1	0.1604	4.48955	13.23	1	2	1
21.6401	17.95	0.1564	4.10665	8.13	1	1	-1
23.0776	31.2	0.1536	3.85399	2.79	1	3	0
24.1017	21.8	0.9595	3.69251	1.57	1	3	3
27.6158	16.08	0.2362	3.2301	24.83	1	1	1
36.2206	10.3	0.2903	2.48006	3.13	1	0	-5
44.8974	14.2	0.0717	2.01888	1.44	2	6	5
51.32	3	0.09	1.78029	2.64	0	2	8
56.88	1	0.09	1.61877	3.19	1	-3	-4
59.3	1	0.09	1.55836	0.56	2	8	2

Table S6: Crystallographic data of C_2.

Pos. [°2Th.]	Height [cts]	FWHM [°2Th.]	d-spacing [Å]	Rel. Int. [%]	**h**	**k**	**l**
11.5666	42.6	0.1574	7.65058	54.19	0	0	2
12.454	72.01	0.1181	7.10736	91.59	0	1	2
14.2804	64.09	0.2362	6.20219	81.51	0	1	0
16.8661	36.54	0.1968	5.25675	46.47	1	0	0
17.3023	36.62	0.1574	5.12518	46.58	1	0	1
19.5302	32.66	0.1968	4.54527	41.54	1	0	2
21.1981	40.74	0.1968	4.19127	51.82	1	-1	-2
23.2063	51.07	0.1181	3.83292	64.96	0	0	4
24.2202	65.65	0.1968	3.6747	83.5	0	1	5
24.9381	43.44	0.1968	3.57053	55.26	1	1	4
25.9935	12.43	0.3149	3.4279	15.81	0	2	1
26.7731	13.47	0.2362	3.32983	17.13	1	-1	-4
27.5036	78.62	0.1968	3.24303	100	1	0	4
29.7969	33.61	0.1574	2.99844	42.75	1	1	-2
31.7232	16.52	0.4723	2.82063	21.02	1	2	5

Table S7: Crystallographic data of C_3.

Pos. [°2Th.]	Height [cts]	FWHM [°2Th.]	d-spacing [Å]	Rel. Int. [%]	**h**	**k**	**l**
10.4499	135.22	0.2755	8.46547	100	0	0	2
11.4571	40.09	0.1181	7.72342	29.65	0	1	2
13.1906	20.45	0.1968	6.71207	15.12	0	1	0
13.8191	25.61	0.2362	6.4082	18.94	0	1	3
16.6328	85.93	0.3149	5.32993	63.55	0	1	-1
18.1969	60.06	0.1968	4.87519	44.42	1	1	2
19.3392	19.87	0.3149	4.58973	14.69	1	1	1
21.1293	9.08	0.3936	4.20475	6.72	1	0	1
23.1285	29.75	0.2362	3.84563	22	1	2	2
24.051	22.61	0.4723	3.70018	16.72	0	2	1
25.8042	18.59	0.3936	3.45262	13.75	1	2	1
27.4571	15.08	0.3149	3.24841	11.15	1	2	7
29.1761	24.86	0.2362	3.06081	18.39	1	3	6
31.7671	16.4	0.3936	2.81684	12.13	0	0	6

Table S8: Crystallographic data of C_4.

Pos. [°2Th.]	Height [cts]	FWHM [°2Th.]	d-spacing [Å]	Rel. Int. [%]	**h**	**k**	**l**
10.4499	135.22	0.2755	8.46547	100	0	0	2
11.4571	40.09	0.1181	7.72342	29.65	0	1	2
13.1906	20.45	0.1968	6.71207	15.12	0	1	0
13.8191	25.61	0.2362	6.4082	18.94	0	1	3
16.6328	85.93	0.3149	5.32993	63.55	0	1	-1
18.1969	60.06	0.1968	4.87519	44.42	1	1	2
19.3392	19.87	0.3149	4.58973	14.69	1	1	1
21.1293	9.08	0.3936	4.20475	6.72	1	0	1
23.1285	29.75	0.2362	3.84563	22	1	2	2
24.051	22.61	0.4723	3.70018	16.72	0	2	1
25.8042	18.59	0.3936	3.45262	13.75	1	2	1
27.4571	15.08	0.3149	3.24841	11.15	1	2	7
29.1761	24.86	0.2362	3.06081	18.39	1	3	6
31.7671	16.4	0.3936	2.81684	12.13	0	0	6

Table S9: IC_{50} values of all the synthesized compounds.

W/V*	C1	C2	C3	C4	HAMQ
	IC_{50} (μM)				
0.5	7.77	8.51	7.92	8.43	16.5
0.25	3.88	4.25	3.96	4.21	8.27
0.125	1.94	2.12	1.98	2.10	4.13
0.0625	0.97	1.06	0.99	1.05	2.06
0.03125	0.48	0.53	0.49	0.52	1.03
0.01562	0.24	0.26	0.24	0.26	0.51
0.00781	0.12	0.13	0.12	0.13	0.25
0.003905	0.06	0.06	0.06	0.06	0.12
0.001952	0.03	0.03	0.03	0.03	0.06
0.000976	0.01	0.01	0.01	0.01	0.03

* W/V – weight by volume. The IC50 is dependent on W/V

Expressions used to calculate global reactivity descriptors

Electronegativity

$$\chi = -{}^{1}/_{2}\,(I + A) \approx {}^{1}/_{2}\,(\varepsilon_L + \varepsilon_L)$$

Global Hardness

$$\eta = (I - A) \approx (\varepsilon_L - \varepsilon_H)$$

Global Electrophilicity

$$\omega = \frac{\chi^2}{2\eta} = \frac{(I + A)^2}{4(I - A)} \approx \frac{(\varepsilon_L + \varepsilon_H)^2}{4(\varepsilon_L - \varepsilon_H)}$$

Electro donating Power

$$\omega^- = \frac{(3I + A)^2}{16(I - A)} \approx \frac{(3\varepsilon_H + \varepsilon_L)^2}{16\eta}$$

Electro accepting Power

$$\omega^+ = \frac{(I + 3A)^2}{16(I - A)} \approx \frac{(\varepsilon_H + 3\varepsilon_L)^2}{16\eta}$$

Net Electrophilicity

$$\Delta\omega^{\pm} = \omega^+ - (-\omega^-) = \omega^+ - \omega^-$$

where ε_H and ε_L are the energies of the HOMO and LUMO, respectively.
